# Neuronal Autophagy Regulates Presynaptic Neurotransmission by Controlling the Axonal Endoplasmic Reticulum

**DOI:** 10.1016/j.neuron.2020.10.005

**Published:** 2021-01-20

**Authors:** Marijn Kuijpers, Gaga Kochlamazashvili, Alexander Stumpf, Dmytro Puchkov, Aarti Swaminathan, Max Thomas Lucht, Eberhard Krause, Tanja Maritzen, Dietmar Schmitz, Volker Haucke

**Affiliations:** 1 Leibniz-Forschungsinstitut für Molekulare Pharmakologie (FMP), 13125 Berlin, Germany; 2 Freie Universität Berlin, Faculty of Biology, Chemistry, and Pharmacy, 14195 Berlin, Germany; 3 Charité Universitätsmedizin Berlin, Corporate Member of Freie Universität Berlin, Humboldt-Universität zu Berlin, and Berlin Institute of Health, 10117 Berlin, Germany

**Keywords:** autophagy, ERphagy, presynapse, neurotransmission, endoplasmic reticulum, calcium, ryanodine receptor

## Abstract

Neurons are known to rely on autophagy for removal of defective proteins or organelles to maintain synaptic neurotransmission and counteract neurodegeneration. In spite of its importance for neuronal health, the physiological substrates of neuronal autophagy in the absence of proteotoxic challenge have remained largely elusive. We use knockout mice conditionally lacking the essential autophagy protein ATG5 and quantitative proteomics to demonstrate that loss of neuronal autophagy causes selective accumulation of tubular endoplasmic reticulum (ER) in axons, resulting in increased excitatory neurotransmission and compromised postnatal viability *in vivo*. The gain in excitatory neurotransmission is shown to be a consequence of elevated calcium release from ER stores via ryanodine receptors accumulated in axons and at presynaptic sites. We propose a model where neuronal autophagy controls axonal ER calcium stores to regulate neurotransmission in healthy neurons and in the brain.

## Highlights

• Neuronal autophagy controls the endoplasmic reticulum (ER) in axons
• Loss of neuronal autophagy leads to increased excitatory neurotransmission
• Increased neurotransmission is due to elevated calcium release from ER stores

## Graphical Abstract

**Figure:**
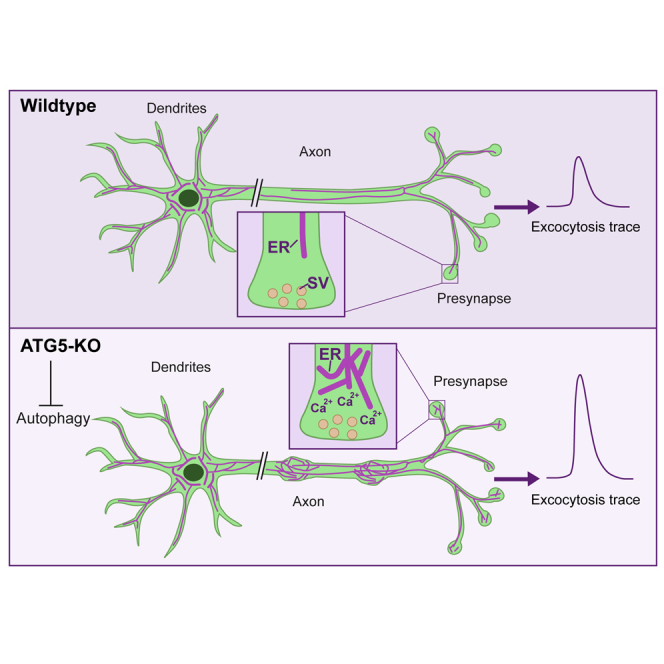

## Teaser

Autophagy is crucial for nervous system function. However, its physiological substrates are largely unknown. Kuijpers et al. demonstrate, using knockout mice conditionally lacking the essential autophagy protein ATG5 and quantitative proteomics paired with electrophysiology and functional imaging experiments, that neuronal autophagy regulates presynaptic neurotransmission by controlling the axonal endoplasmic reticulum.

## Introduction

Information processing in the brain critically relies on the relay of information from a presynaptic neuron to the postsynapse via regulated neurotransmitter release. This process is triggered by the action potential (AP)-triggered, calcium-driven exocytic fusion of neurotransmitter-containing synaptic vesicles (SVs) at active zone (AZ) release sites (Jahn and Fasshauer, 2012; Südhof, 2013). Exocytic SV fusion is followed by endocytosis of SV membranes and reformation of functional SVs to replenish the SV pool (Haucke et al., 2011; Murthy and De Camilli, 2003; Rizzoli, 2014). The efficacy of neurotransmitter release is modulated by presynaptic calcium influx via voltage-sensitive calcium channels located at AZs, calcium efflux and sequestration (Nanou and Catterall, 2018; Neher and Sakaba, 2008), as well as calcium-induced calcium release from internal endoplasmic reticulum (ER) stores located in the axon and at presynaptic sites (Bezprozvanny and Kavalali, 2020; Galante and Marty, 2003; Irie and Trussell, 2017).

Because neurons are long-living postmitotic cells, the majority of their synapses need to be maintained for the entire lifespan of the organism (Cajigas et al., 2010). To prevent neuronal and synaptic dysfunction, neurons have evolved mechanisms for removal of toxic or defective proteins and organelles to maintain regulated neurotransmission and the integrity of their functional proteome. Among these mechanisms are lysosomal turnover of membrane proteins and autophagy, a cellular process by which defective proteins and organelles are degraded through sequestration in autophagosomes and delivery to lysosomes (Hill and Colón-Ramos, 2020; Nikoletopoulou and Tavernarakis, 2018; Vijayan and Verstreken, 2017). In neurons, autophagy has been implicated in diverse processes ranging from development, including signaling via neurotrophins (Andres-Alonso et al., 2019; Kononenko et al., 2017), to pathogenesis of neurodegenerative disorders (Moreau et al., 2014; Nixon, 2013; Ravikumar et al., 2010; Sarkar et al., 2007; Stavoe and Holzbaur, 2019). The importance of the autophagy system in the brain is emphasized by the fact that knockout of core ATG proteins, such as autophagy-related protein 5 (ATG5) or ATG7, induces accumulation of non-degraded protein aggregates, neurodegeneration, and neuronal cell death in mice (Hara et al., 2006; Komatsu et al., 2006, 2007). Conversely, induction of autophagy counteracts neurodegeneration in disease models (Moreau et al., 2014; Nixon, 2013; Ravikumar et al., 2004, 2010; Williams et al., 2006).

Despite the general importance of autophagy for neuronal viability and function (Friedman et al., 2012; Hill and Colón-Ramos, 2020; Nikoletopoulou and Tavernarakis, 2018; Vijayan and Verstreken, 2017), the physiological substrates of neuronal autophagy and the mechanisms by which defects in neuronal autophagy affect neuronal and synaptic function are largely unknown. Autophagosomes are formed in distal axons (Hill and Colón-Ramos, 2020; Maday and Holzbaur, 2014; Maday et al., 2012) and in the presynaptic compartment (Azarnia Tehran et al., 2018; Murdoch et al., 2016; Soukup et al., 2016; Soukup and Verstreken, 2017). Distally formed autophagosomes mature during their retrograde axonal transport (Guedes-Dias and Holzbaur, 2019; Stavoe and Holzbaur, 2019) prior to their fusion with degradative lysosomes enriched in proximal axons and in neuronal somata (Hill and Colón-Ramos, 2020; Maday and Holzbaur, 2014; Maday et al., 2012). In addition to this largely constitutive process of neuronal autophagy (Maday and Holzbaur, 2016), formation of autophagosomes has been suggested to be facilitated by mitochondrial damage (Ashrafi et al., 2014), neuronal activity (Shehata et al., 2012; Wang et al., 2015), overexpression of aggregation-prone proteins (Corrochano et al., 2012), reactive oxygen species (ROS)-induced protein oxidation (Hoffmann et al., 2019), or genetic depletion of key AZ proteins (Okerlund et al., 2017).

We demonstrate, using knockout mice conditionally lacking the essential autophagy protein ATG5 and quantitative proteomics, that loss of neuronal autophagy causes selective accumulation of tubular ER in axons, resulting in increased excitatory neurotransmission because of elevated calcium release from ER stores via ryanodine receptors. Our findings suggest that neuronal autophagy controls axonal ER calcium stores to regulate neurotransmission in healthy neurons and in the brain.

## Results

### Selective Loss of Neuronal Autophagy in the Absence of ATG5 Facilitates Excitatory Neurotransmission and Causes Premature Death *In Vivo*

**Figure 1:**
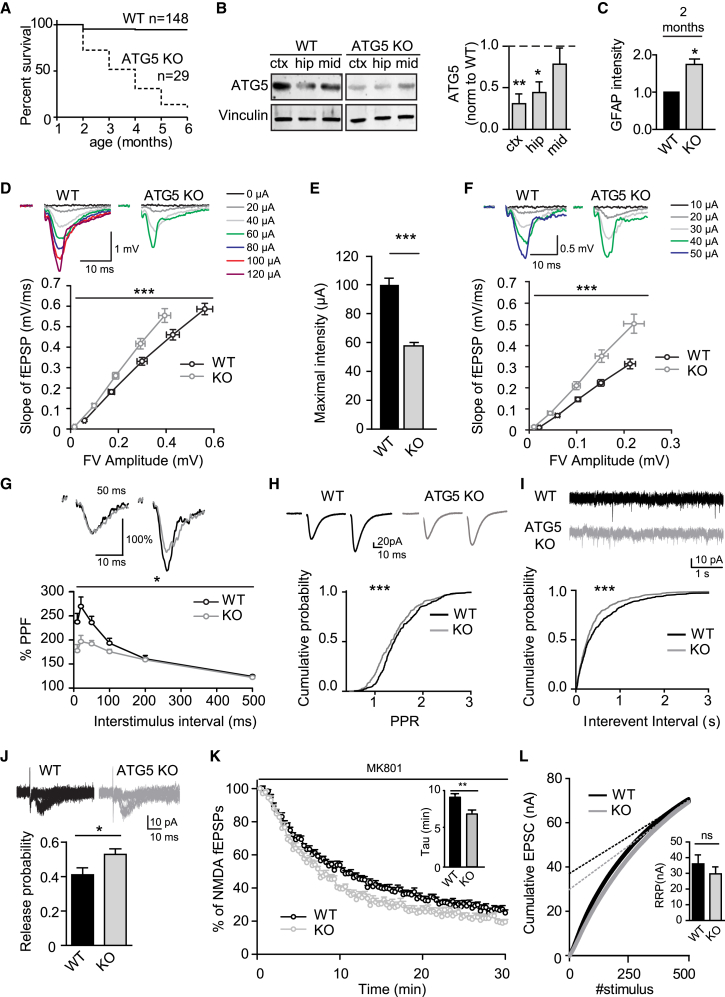
Selective Loss of Neuronal Autophagy Facilitates Excitatory Neurotransmission (A) Decreased survival of KO mice conditionally deleted for ATG5 by transgenic expression of Cre recombinase under the telencephalon-specific EMX promoter (ATG5^flox/flox^; EMX1-Cre). (B) Western blot and quantification showing an ATG5 decrease primarily in the cortex (ctx) and hippocampus (hip) of 2-month-old ATG5-cKO mice. n = 4 mouse pairs for ctx and midbrain (mid) and n = 3 mouse pairs for hip, one-sample t test. (C) Quantification of GFAP immunostaining in 6- to 7-week-old control and ATG5-cKO brain slices. Slices were taken from 3 mice; one-sample t test. See also Figure S1C. (D) Basal excitatory neurotransmission measured as the relationship between fiber volley (FV) amplitudes and slopes of fEPSPs in WT control (n = 24 slices, 12 mice) and ATG5-cKO (n = 24 slices, 12 mice) mice. Representative fEPSP traces (above) and quantified data are shown. Significant difference between WT control and ATG5-cKO slices encompassing the curve; two-way repeated-measures ANOVA. (E) A lower stimulation intensity is required to elicit maximal responses in ATG5-cKO (58.8 ± 2.1 μA) compared with control mice (100.8 ± 4.6 μA); t test. (F) Basal excitatory neurotransmission measured as relationships between FV amplitudes and slopes of fEPSPs in WT control (n = 11 slices, 6 mice) and ATG5-cKO (n = 10 slices, 6 mice) mice in the presence of the GABA_A_ receptor antagonist picrotoxin (50 μM). Representative fEPSP traces (above) and quantified data are shown. Significant difference between WT control and ATG5-cKO slices encompassing the curve; two-way repeated-measures ANOVA. (G) Measurements of paired-pulse facilitation (PPF) in the presence of the GABA_A_ receptor antagonist picrotoxin (50 μM) reveal significantly reduced PPF in ATG5-cKO (n = 10 slices, 6 mice) compared with control (n = 11 slices, 6 mice) mice. Representative traces of PPF at a 50-ms interstimulus interval (above) and quantified data over a range of interstimulus intervals (10–500 ms), given as a percentage of the second in relation to the first response (percent PPF), show reduced facilitation of the second response in ATG5-cKO mice; two-way repeated-measures ANOVA. (H) Cumulative probability shows a left-shifted distribution for PPR in ATG5-cKO mice. n = 28 (WT) or 30 (KO) slices from 8 animals; Kolmogorov-Smirnov test. (I) Cumulative probability distribution shows decreased interevent intervals for sEPSCs in ATG5-cKO mice. n = 17 (WT) or 21 (KO) cells from 7 and 6 animals, respectively; Kolmogorov-Smirnov test. (J) Release probability evaluated by a minimal stimulation protocol shows increased release probability (i.e., decreased failure rate) in ATG5-cKO mice. n = 23 (WT) or 24 (KO) cells from 5 animals; t test. (K) Release probability evaluated by NMDA receptor-mediated fEPSP amplitude decay. Averaged NMDA receptor-mediated amplitudes in the presence of MK801 (30 μM) show significantly faster decay in KO mice (see tau values in the bar graph). n = 12 (WT) or 10 (KO) slices from 7 and 6 animals, respectively; t test. See also Figures S2B and S2C. (L) Estimation of RRP size by back-extrapolation (last 50 data points) of the cumulative EPSC to the y axis. n = 13 (WT) or 15 (KO) cells from 4 animals; Mann-Whitney test. All data show mean ± SEM. ns, not significant; ^∗^p < 0.05, ^∗∗^p < 0.01, ^∗∗∗^p < 0.001.

It has been demonstrated previously that early loss of ATG5 in neurons and glial cells throughout the nervous system causes progressive motor deficits and severe neurodegeneration associated with ubiquitin-containing cytoplasmic inclusions (Hara et al., 2006; Komatsu et al., 2006). To determine the physiological consequences of selective ablation of autophagy in neurons in the neocortex and hippocampus, we crossed ATG5^*flox/flox*^ mice with a transgenic EMX1-*Cre* line that expresses Cre recombinase in postmitotic excitatory neurons of the cortex and hippocampus. Conditional ATG5^*flox/flox*^; EMX1-*Cre* knockout (KO) mice (hereafter called ATG5-conditional knockout [cKO]) were born at normal Mendelian ratios (Figure S1A) but displayed reduced postnatal growth (Figure S1B) and early postnatal lethality between 2 and 6 months of age (Figure 1A). Analysis by immunoblotting revealed profound loss of ATG5 protein mainly in the cerebral cortex and in the hippocampus (Figure 1B). This was accompanied by accumulation of the autophagy adaptor and substrate protein p62 (elevated 3.9 ± 2.6-fold in the cortex, 2.6 ± 1.2-fold in the hippocampus, and 2.1 ± 0.5-fold in the midbrain, as measured by quantitative immunoblotting), consistent with prior observations in ATG5^*flox/flox*^; nestin-*Cre* KO mice lacking ATG5 throughout the brain (Hara et al., 2006). Accumulation of p62 in the cortex and hippocampus as well as signs of astrogliosis were also observed by confocal imaging in brain slices (Figures 1C and S1C). Moreover, caspase activity was elevated in aged 4-month-old but not in young ATG5-cKO mice (Figures S1D and S1E). No significant alterations in the levels of key presynaptic (i.e., Synaptotagmin 1 and Synaptobrevin 2) and postsynaptic proteins (i.e., Homer 1) (Figures S1F and S1G), the number of vGLUT1/Homer 1-containing excitatory synapses (Figures S1H and S1I), or synapse density analyzed ultrastructurally by electron microscopy (Figures S1J and S1K), were observed. These results show that loss of neuronal autophagy impairs postnatal viability and causes neuronal cell death in mice *in vivo* but does not significantly alter synapse number or density.

To analyze whether and how loss of neuronal autophagy in the conditional absence of ATG5 in excitatory neurons affects synaptic transmission, we recorded field excitatory postsynaptic potentials (fEPSPs) of CA3-CA1 synapses in acute hippocampal slices. These measurements revealed elevated basal synaptic transmission in ATG5-cKO mice. The slopes of fEPSPs over fiber volley (FV) amplitudes were increased significantly (Figure 1D), and lower stimulation intensities were required to elicit maximal responses in ATG5-cKO slices (Figure 1E). Moreover, elevated fEPSP slopes over FV amplitudes were also observed in the presence of the GABA_A_ receptor antagonist picrotoxin (Figure 1F), suggesting that elevated excitatory transmission in ATG5-cKO slices was not a consequence of impaired synaptic inhibition. We therefore followed the alternative hypothesis that loss of neuronal autophagy facilitates excitatory neurotransmission by increasing presynaptic release probability (Branco and Staras, 2009). Slices from ATG5-cKO mice showed reduced paired-pulse facilitation (PPF) of fEPSPs, a surrogate measure of presynaptic release probability (Branco and Staras, 2009), in the presence of picrotoxin (Figure 1G). Significantly reduced PPRs of evoked excitatory postsynaptic currents (eEPSCs) were also observed in patch-clamp recordings (Figure 1H). Moreover, conditional loss of ATG5 led to a significant increase in the frequency (Figure 1I) but not amplitude (Figure S2A) of spontaneous EPSCs (sEPSCs). The increased presynaptic release probability of ATG5-cKO hippocampal synapses was further confirmed by patch-clamp recordings using a minimal stimulation protocol (Figure 1J) and by measuring the decay of N-methyl-D-aspartate (NMDA) receptor-mediated fEPSP amplitudes in the presence of the use-dependent NMDA receptor antagonist MK-801 (Weisskopf and Nicoll, 1995; Figures 1K, S2B, and S2C). In contrast, the NMDA/α-amino-3-hydroxy-5-methyl-4-isoxazolepropionic acid (AMPA) ratio (Figure S2D) and the size of the readily releasable SV pool determined by back-extrapolation of the cumulative EPSCs to the y axis (Figure 1L) were unaffected in slices from ATG5-cKO mice. We conclude that elevated excitatory neurotransmission in ATG5-cKO mice is a presynaptic phenotype that does not appear to be caused by impaired synaptic inhibition. These data are also consistent with the fact that selective loss of ATG5 in postsynaptic neurons does not alter excitatory neurotransmission (Shen et al., 2020).

Next we wanted to find out whether the observed synaptic phenotype is specific for hippocampal CA1 synapses or represents a more general phenotype. To this aim, we investigated a very different synaptic connection, the hippocampal mossy fiber (mf) synapse, which has a number of specific features; e.g., low basal release probability, pronounced frequency facilitation, and a presynaptic form of long-term potentiation that lacks NMDA receptor involvement (see Nicoll and Schmitz, 2005, for a review). In addition, use-dependent amplification of presynaptic Ca^2+^ signaling by axonal ryanodine receptors has been postulated (Shimizu et al., 2008). Previous work has established a close causal link between presynaptic release probability and synaptic plasticity, including long-term potentiation (LTP) at mf CA3 synapses (Nicoll and Schmitz, 2005; Schulz, 1997; Sola et al., 2004; Weisskopf and Nicoll, 1995; Yang and Calakos, 2013; Zucker and Regehr, 2002). Hence, we probed presynaptic forms of short- and long-term plasticity and observed decreased post-tetanic potentiation (PTP) and blockade of LTP at hippocampal mf synapses from ATG5 KO mice (Figures S2E–S2G). These combined data indicate that loss of neuronal autophagy in the absence of ATG5 causes gain of synaptic neurotransmission and loss of presynaptic plasticity at glutamatergic synapses in areas CA1 and CA3 of the hippocampus.

**Figure 2:**
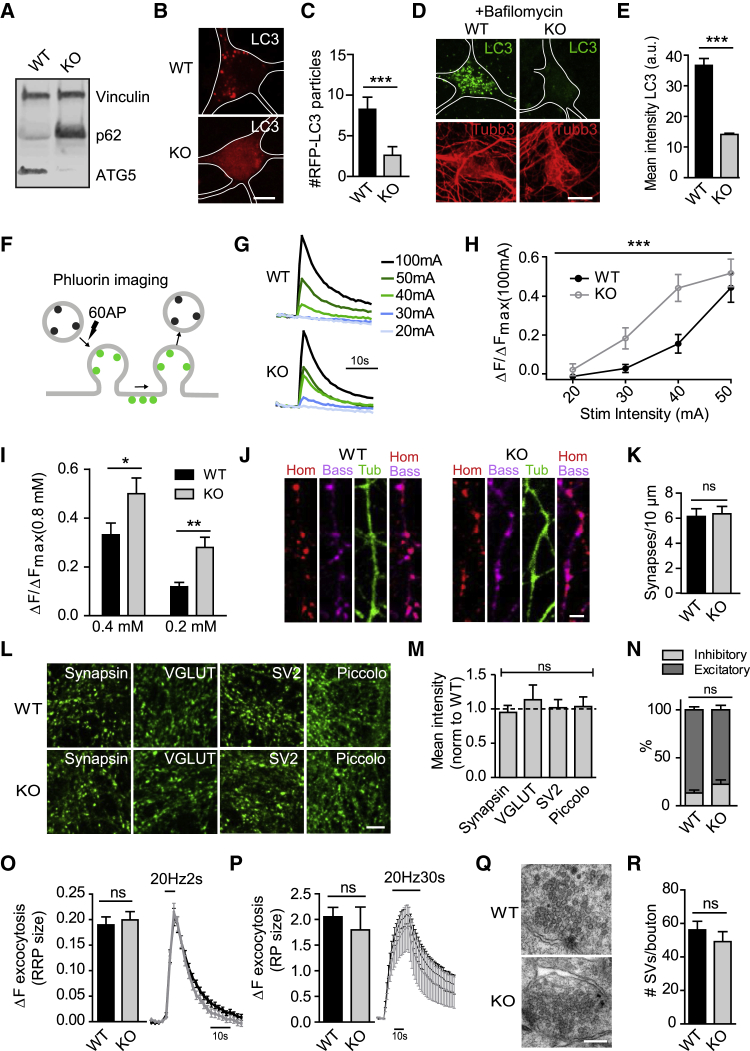
ATG5-iKO Hippocampal Neurons Display Increased Stimulation-Dependent SV Release (A) Immunoblot showing ATG5 decrease and p62 increase in lysates from tamoxifen-inducible ATG5-iKO (KO) hippocampal cultures. (B and C) Representative images (B) of hippocampal WT and KO hippocampal neurons expressing mRFP-LC3. Note the decreased LC3 punctum numbers in ATG5 KO neurons; quantified in (C). Scale bar, 10 μm. n = 20 cells from a representative experiment; Mann-Whitney test. (D and E) ATG5 KO hippocampal neurons show deficient LC3-positive punctum formation upon bafilomycin treatment (10 nm, 4 h). Representative immunofluorescence images show LC3 staining in (D) (quantified in E). Scale bar, 10 μm. n = 42 cells, 1 experiment; Mann-Whitney test. (F–H) Detection of exocytosis using Synaptophysin-pHluorin. (F) Schematic showing reporter de-acidification during vesicle fusion with the plasma membrane. (G) Example traces (averaged from a representative experiment) showing a stimulus-dependent decrease in pHluorin signal in WT and KO hippocampal synapses. (H) Graph showing mean peak fluorescence upon different stimulation intensities. Values per cell are normalized to the corresponding maximal fluorescent peak at 100 mA (Fmax). n = 17–35 cells, 20 boutons per cell, 5 independent experiments; two-way ANOVA. (I) Graph showing mean peak fluorescence of the pHluorin signal under conditions of different extracellular calcium concentrations. Values per cell are normalized to the corresponding Fmax at 0.8 mM calcium. n = 21 cells, 20 boutons per cell, 3 independent experiments; t test. (J) Representative confocal images of hippocampal neurons immunostained for β3-tubulin (green), Homer 1 (postsynaptic, red), and Bassoon (presynaptic, magenta). Scale bar, 2 μm. (K) Synapse numbers in WT and KO cultures expressed as the number of Homer 1/Bassoon-positive puncta along β3-tubulin-positive neurite length. n = 3 independent experiments, ~2,900 synapses per genotype; paired t test. (L) Representative confocal images of hippocampal neurons immunostained for Synapsin-1, VGLUT1, SV2, and Piccolo. Scale bar, 5 μm. (M) Quantification of Synapsin-1, VGLUT1, SV2, and Piccolo immunostaining intensities. The mean values for the control are set to 1, and the mean value for the KO is expressed relative to this. n = 3 independent experiments, 26-37 images per condition; one-sample t test. (N) Percentage of inhibitory and excitatory synapses in WT and KO hippocampal cultures determined by Synapsin (marker for all synapses) and vGAT (inhibitory synapse marker) antibody staining. Excitatory synapses are Synapsin positive and vGAT negative. n = 3 independent experiments, 45–47 images per condition; paired t test. (O) Quantification and average traces of Synaptophysin-pHluorin-expressing neurons stimulated with 40 APs (20 Hz) to determine the size of the readily releasable SV pool (RRP). n = 3 independent experiments, 20 cells per condition; paired t test. (P) Quantification and average traces of Synaptophysin-pHluorin-expressing neurons stimulated with 600 APs (20 Hz) to determine the size of the recycling SV pool (RP). n = 3 independent experiments, 20–24 cells per condition; paired t test. (Q and R) Representative electron micrographs of nerve terminals in WT and KO hippocampal cultures show no difference in the number of SVs per bouton (quantified in R). Scale bar, 1 μm. n = 41 (WT) and 45 (KO) boutons, 1 experiment; Mann-Whitney test. All data represent mean ± SEM. ^∗^p < 0.05, ^∗∗^p < 0.01, ^∗∗∗^p < 0.001.

We challenged these unexpected findings in slices by optical imaging experiments in cultured neurons. We crossed ATG5^*flox/flox*^ mice with a transgenic CAG-*iCre* line in which Cre recombinase activity is under tamoxifen control. We then prepared primary neurons from the hippocampus of these ATG5^*flox/flox*^; CAG-*iCre* mice (referred to as ATG5-inducible knockout [iKO] hereafter) and corresponding wild-type (WT) mice and treated them with tamoxifen to acutely disrupt the ATG5 gene. As expected, tamoxifen-induced conditional loss of ATG5 in hippocampal neurons (Figure 2A) abrogated formation of LC3-containing autophagosomes (Figures 2B and 2C; see Figure S3C for inhibitory neurons), a phenotype most prominently observed following application of the vacuolar ATPase (v-ATPase) blocker bafilomycin (Figures 2D and 2E). As expected, blockade of neuronal autophagy in the absence of ATG5 was accompanied by progressive accumulation of the established autophagy substrate protein p62 (Figures 2A, S3A, and S3B) in neuronal somata. To study the effects of defective autophagy in the absence of ATG5 on presynaptic function, we monitored SV exo-endocytosis using pH-sensitive pHluorin as a reporter (Kavalali and Jorgensen, 2014; Figure 2F). Synaptophysin-pHluorin-expressing hippocampal neurons from WT or ATG5-iKO mice were stimulated with 60 APs at different stimulation intensities, and SV exo-endocytosis was monitored by optical imaging (Figure 2G). Similar stimulation intensities induced Synaptophysin-pHluorin responses with significantly higher amplitudes in ATG5-iKO neurons (Figures 2H and S3D), akin to our electrophysiological data from acute slice preparations (compare with Figure 1D). Moreover, ATG5-cKO neurons displayed increased calcium sensitivity of neuroexocytosis (Figures 2I, S3E, and S3F). Akin to our results from ATG5-cKO mice, no changes in synapse density (Figures 2J and 2K) or in the levels or localization of SVs (i.e., Synapsin 1, SV2, vesicular glutamate transporter [vGLUT1]) and AZ proteins (i.e., Piccolo) were detected (Figures 2L and 2M). Acute genetic loss of neuronal autophagy in ATG5-iKO neurons did not affect the ratio of excitatory versus inhibitory synapses (Figure 2N), the readily releasable or total recycling vesicle pool sizes (Figures 2O and 2P), or the total SV pool size determined at the ultrastructural level (Figures 2Q and 2R).

In summary, loss of neuronal autophagy causes cell-autonomous facilitation of presynaptic neurotransmission (Figures 1, 2, and S2) that is not explained by alterations in the number or density of synapses (Figures S1F–S1K, 2J, and 2K), the excitatory versus inhibitory synapse ratio (Figure 2N), or presynaptic vesicle numbers, pool sizes, and SV localization (Figures 2L, 2M, and 2O–2R).

### Accumulation of Axonal Tubular ER Induced by Blockade of Neuronal Autophagy in the Absence of ATG5

**Figure 3:**
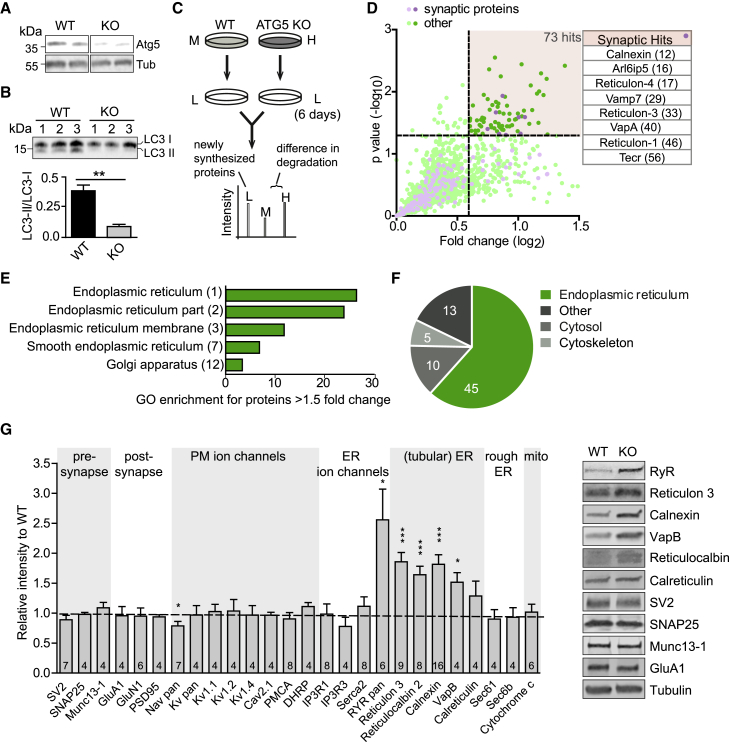
Decreased Degradation and Accumulation of ER Proteins in ATG5-iKO Neurons (A) Immunoblots showing ATG5 decrease in cell lysates from ATG5-iKO cerebellar granule neuron (CGN) cultures compared with control culture lysates. (B) Analysis of the LC3-II/LC3-I ratio in immunoblots of CGN lysates from WT and KO cultures. n = 3; unpaired t test. (C) Schematic showing the pulsed SILAC procedure to measure protein degradation. CGN cultures were grown for 2 weeks in medium containing heavy (H) or medium (M) variants of lysine and arginine. On day 14, the medium was replaced with normal medium containing unlabeled (L) amino acids. After 0 (t = 0) or 6 (t = 6) days, cells were harvested, mixed, and analyzed by MS analysis, resulting in a list of H/M ratios for each protein. The example shows an H-labeled peptide that is degraded at slower rates than the M-labeled peptide, resulting in H/M ratios greater than 1. (D) Four separate experiments were performed, in which 1,753 proteins were identified that exhibited H/M (KO/WT) ratios in at least 3 experiments (and 2 conditions, t = 0 and t = 6). Of the 1,753 proteins, 180 are considered to be synaptic proteins. To evaluate protein degradation over the course of 6 days, ratios at t = 6 are divided by t = 0 ratios, and fold changes are plotted. Of 1,753 proteins, 73 showed a significant increase in average H/M ratios over the period of 6 days (defined as log_2_fold change > 0.6 and p < 0.05, dotted lines); that is, they exhibited slower degradation rates in ATG5-iKO neurons. The table shows the protein hits considered to be synaptic (the rank of hit is shown in brackets). (E) Gene Ontology analysis indicates that most of the proteins that show slower degradation rates in KO neurons (fold change > 1.5) are localized to the ER (the rank of overrepresented GO Cellular component is shown in brackets). (F) Main subcellular localization of the 73 hit proteins (UniProtGO Annotation Database). (G) Immunoblot analysis and representative examples of lysates from WT and KO CGNs in culture, using antibodies against the indicated proteins. Bars show the protein level change of the indicated proteins normalized to the housekeeping gene tubulin. The mean values for the controls are set to 1. n, indicated in bars; one-sample t test. All data represent mean ± SEM. ^∗^p < 0.05, ^∗∗^p < 0.01, ^∗∗∗^p < 0.001.

**Figure 4:**
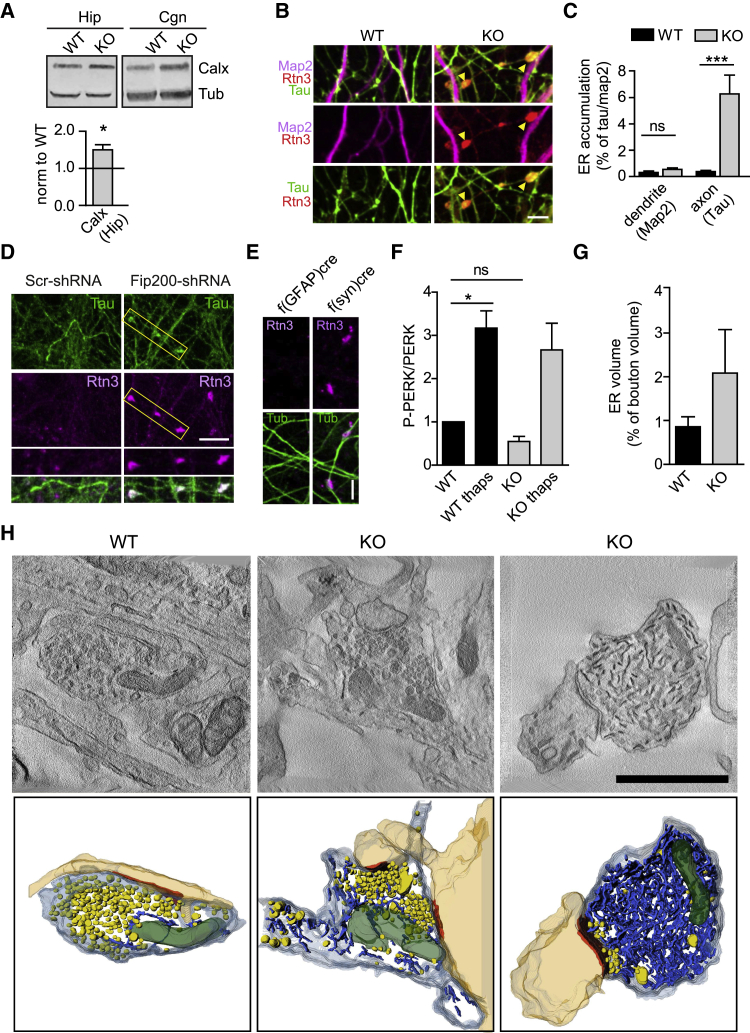
Inhibition of Neuronal Autophagy Leads to Accumulation of Smooth ER Proteins and Tubules in Hippocampal Axons (A) Elevated levels of ER membranes monitored by Calnexin in lysates from hippocampal neurons in culture, similar to what is seen in lysates from CGNs (see also Figure 3G). Samples were analyzed by immunoblotting. The bar displays Calnexin protein levels normalized to tubulin as a control. Data for the WT were set to 1. n = 7 independent experiments; one-sample t test. (B) Representative confocal images of WT and ATG5-iKO hippocampal neurons immunostained for the ER marker Reticulon 3 (Rtn3), the axonal marker Tau, and the dendritic marker Map2. Yellow arrows indicate ER accumulations in KO axons. Scale bar, 5 μm. (C) Quantification of Rtn3 in WT and KO hippocampal neurons, expressed as dendritic (Map2) or axonal (Tau) area covered with Rtn3 accumulations. n = 50 images, 4 independent experiments; Mann-Whitney test. (D) Representative confocal images of control hippocampal neurons and neurons transduced with Fip200-shRNA immunostained for the ER marker Rtn3 and the axonal marker Tau. ER accumulates in Fip200 KD axons. Scale bar, 10 μm. (E) Cre-mediated ATG5 depletion in hippocampal neurons (f(syn)cre) but not in astrocytes (f(gfap)cre) leads to neuronal Rtn3 accumulation. Scale bar, 10 μm. (F) Quantification of PERK phosphorylation (p-PERK) in WT and ATG5-iKO lysates from CGNs in culture treated with thapsigargin (thaps) to induce ER stress (1 μM, 16 h) or left untreated. The P-PERK/PERK ratio observed in WT lysates was set to 1. n = 4 independent experiments; one-sample t test. See also Figures S4M and S4N for representative blots and P-JNK quantification. (G and H) 3D analysis of ER tubules in synaptic terminals. (G) Bars indicate the average ER volume in WT and ATG5-iKO boutons. n = 12 tomographic reconstructions, 12 boutons per group. (H) Single virtual sections and 3D transmission electron microscopy (TEM) tomography reconstructions of synaptic boutons showing postsynaptic densities (orange), ER tubules (blue), SVs (yellow), and mitochondria (green). Examples show a WT bouton and two ATG5-iKO boutons with medium and severe ER volume increases. Scale bar, 1 μm. All data represent mean ± SEM. ^∗^p < 0.05, ^∗∗∗^p < 0.001.

Because enhanced excitatory neurotransmission did not appear to be caused by accumulation of presynaptic exo- or endocytic proteins or SVs, we conducted an unbiased quantitative proteomics analysis of the steady-state levels and turnover of neuronal proteins in WT versus ATG5-iKO neurons to identify factors that might conceivably regulate neurotransmission. Because hippocampal neurons in culture require the presence of astrocytes and are limited in number, we resorted to cerebellar granule neurons (CGNs), which can be cultured in the absence of other cell types. WT or ATG5-iKO CGNs were treated with tamoxifen to induce ATG5 loss (Figure 3A), resulting in blockade of autophagosome formation, as evidenced by defective conversion of the key autophagy component LC3 from its inactive LC3-I to the active LC3-II isoform (Figure 3B). We then conducted quantitative proteomics analysis of neuronal protein turnover by stable isotope labeling with amino acids in cell culture (SILAC) experiments. CGNs were grown in medium containing heavy or medium variants of lysine and arginine for 14 days and analyzed directly by tandem mass spectrometry (MS/MS) to determine their steady-state levels or pulsed for a further 6 days in medium containing light (i.e., unlabeled) amino acids before MS/MS analysis (Figure 3C). Of the 1,753 proteins identified in at least 3 of 4 experiments (Table S1), 73 proteins exhibited a reduced degradation rate, as evidenced by a significantly increased ratio of heavy (KO)- to-medium (WT)-labeled peptides (H/M ratio) over the 6 day-period (i.e., increased (H/M) t = 6/(H/M) t = 0), including several allegedly synaptically localized (Hakim et al., 2016) ER membrane proteins (i.e., Reticulon-1, Reticulon-4, VapA, and Calnexin) (Figure 3D; Table S1). Many of these factors already displayed increased levels at steady state (Figures S3G and S3H). Further gene ontology analysis indicated that the majority of proteins with reduced turnover in the absence of ATG5-mediated neuronal autophagy were proteins known to be localized to the ER (Berner et al., 2018; Saheki and De Camilli, 2017; Westrate et al., 2015) with a preference for tubular ER membrane proteins (Figures 3E and 3F). To confirm these data with an independent approach, we determined the steady-state levels of distinct classes of ER membrane proteins (i.e., tubular versus rough/sheet ER) by quantitative immunoblot analysis of CGN neurons in culture. This analysis revealed a prominent accumulation of tubular ER membrane proteins, such as Reticulon 3, VapB, and the ryanodine receptor (RyR), an ER-localized, ligand-gated calcium channel (Del Prete et al., 2014; see Figures 3G and S3I for reduced degradation rates; because of its large size and the resulting poor migration behavior in SDS-PAGE, RyR could not be detected in all MS/MS experiments). Lumenal ER proteins, such as Reticulocalbin and Calreticulin, accumulated moderately, whereas no change in the levels of rough ER membrane proteins, such as Sec61and Sec61b, involved in secretory protein synthesis, was detectable (Figure 3G). Strikingly, we observed no change in the levels of presynaptic vesicle (i.e., SV2) and AZ proteins (i.e., Munc13-1); postsynaptic (i.e., GluA1 and GluN1) and plasma membrane ion channels, including voltage-gated calcium (i.e., Cav2.1) or K^+^ channels (i.e., Kv1.1 and Kv1.2) and their associated factors; or mitochondrial cytochrome *c* (Figure 3G). Accumulation of ER proteins, such as Calnexin, was also observed in hippocampal neurons in culture (Figure 4A; see also further below), suggesting that autophagy-mediated turnover of tubular ER is a general feature of central nervous system (CNS) neurons. These data indicate that the tubular ER is a major substrate for neuronal autophagy mediated by ATG5 in healthy unperturbed CNS neurons in the absence of proteotoxic challenge.

Previous work using live imaging has established that autophagosomes form preferentially in distal axons and at presynaptic sites (Hill and Colón-Ramos, 2020; Maday and Holzbaur, 2014; Maday et al., 2012) via a largely constitutive mechanism (Maday and Holzbaur, 2016) that depends on ATG5. We therefore studied whether the accumulation of tubular ER detected at the proteomic level in cultured cerebellar or hippocampal neurons (Figures 3 and 4A) was homogeneous throughout the neuron or specific to axons versus the neuronal soma or dendrites. Confocal imaging of hippocampal neurons from tamoxifen-treated ATG5-iKO mice revealed a pronounced accumulation of tubular ER marked by Reticulon 3 in Tau-positive/MAP2-negative axons (Figures 4B and 4C), whereas tubular ER levels were altered insignificantly in dendrites and neuronal somata (Figures S4A and S4B). Axons of cultured inhibitory hippocampal neurons marked by GAD6 also displayed tubular ER accumulation (Figure S4C). In ATG5-iKO neurons, the axonal ER often appeared as distinct varicosities, possibly representing accumulated ER tubules (Figure 4B and below). Similar results were seen when the ER was marked by Calnexin (Figure S4D) or upon transfection with DsRed-KDEL, a probe for the ER lumen (Figures S4E and S4F). Loss of ATG5 in astrocytes did not result in accumulated ER in axons (Figure 4E), indicating that the observed neuronal ER phenotype is cell autonomous. ER-containing axonal varicosities were clearly distinct from p62-positive ubiquitin conjugates detected mostly in neuronal cell bodies of ATG5-iKO neurons (Figure S4G). To verify that the tubular ER accumulation in axons is indeed a consequence of perturbed neuronal autophagy rather than a phenotype unique to ATG5 loss, we acutely blocked neuronal autophagy by inhibiting VPS34, a phosphatidylinositol 3-phosphate-synthesizing lipid kinase required for the early steps of autophagy (Ariosa and Klionsky, 2016; Ravikumar et al., 2008; Vijayan and Verstreken, 2017). Acute pharmacological inhibition of VPS34 by an established specific small-molecule inhibitor, VPS34-IN1 (Bago et al., 2014; Ketel et al., 2016), phenocopied genetic loss of ATG5 with respect to accumulation of tubular ER in axons (Figure S4H). Moreover, tubular ER marked by Reticulon 3 also accumulated in Tau-positive axons, often as punctate varicosities, in hippocampal neurons depleted of the early-acting autophagy protein FIP200 by lentiviral knockdown (Figure 4D). In contrast, loss of ATG5 did not affect the levels or localization of the Golgi complex or LAMP1-containing late endosomes/lysosomes (Figure S4I), rough ER marked by Sec61b (Figure S4J), or mitochondria (Figures S4K and S4L). In spite of the pronounced accumulation of axonal ER, no signs of induction of the ER stress response probed by specific antibodies against the active phosphorylated form of the ER stress-induced kinases protein kinase R-like endoplasmic reticulum kinase (PERK) or jun N-terminal kinase (JNK) were detectable in ATG5-iKO neurons (Figures 4F, S4M, and S4N). Moreover, ER tubule diameter, a surrogate measure of ER stress (Schuck et al., 2009; Zhang and Hu, 2016), analyzed by electron microscopy (EM), was unchanged in ATG5-iKO hippocampal neurons (Figure S4O).

The data so far suggest that blockade of neuronal autophagy in the absence of ATG5 causes accumulation of tubular ER in axons and, possibly, at synapses. We further probed this hypothesis at the ultrastructural level by electron tomography. Tomographic analysis of hippocampal neurons in culture confirmed the dramatic accumulation of ER tubules in axons and at presynaptic sites (Figures 4G and 4H). Elevated numbers of ER tubules were observed at ATG5-iKO boutons (Figures 4G and 4H, center panels). In some cases, presynaptic boutons were filled with ER tubules (Figure 4H, right panels), suggesting that neuronal autophagy is preferentially active in a subset of nerve terminals and/or distal axons.

Our findings show that blockade of neuronal autophagy in the absence of ATG5 causes pronounced accumulation of tubular ER in axons and at presynaptic sites, whereas the core machinery for neurotransmission and SV exo-endocytosis appears to be unperturbed.

### Accumulation of Tubular ER in Axons of ATG5 KO Neurons Is Caused by Selective Blockade of Autophagy/Lysosome-Mediated Turnover of ER Membranes

**Figure 5:**
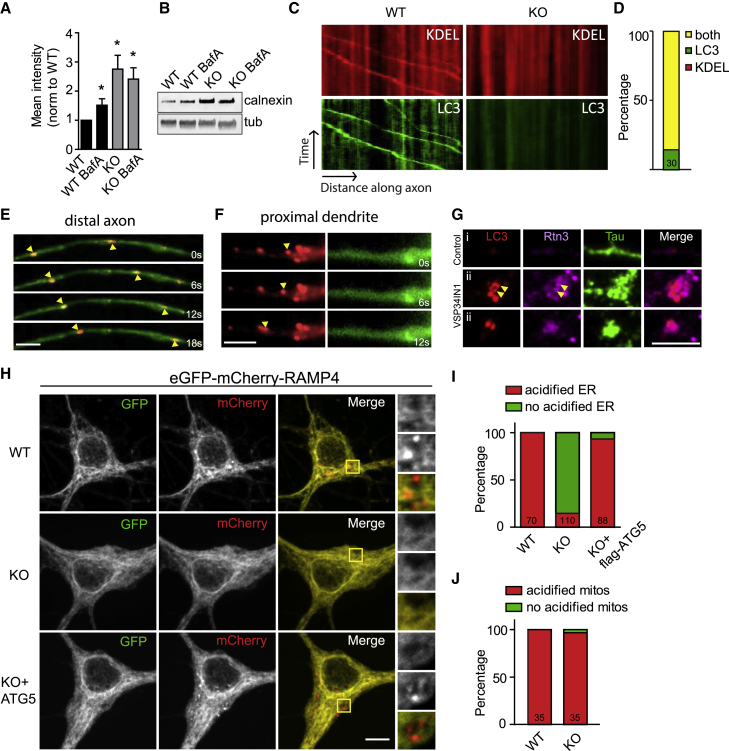
Neuronal ER Co-traffics with Axonal Autophagosomes and Is Degraded in Lysosomes (A and B) Inhibiting autophagy by preventing vacuolar acidification in the presence of bafilomycin A1 causes Calnexin accumulation. (A) Quantification of immunoblots of WT and ATG5-iKO CGN cultures treated with bafilomycin A1 (BafA, 2 nM, 24 h). All conditions are compared with the WT; values for WT were set to 1. n = 7 (WT, WT BafA, KO) or 6 (KO BafA) independent experiments; one-sample t test. (B) Representative immunoblots of lysates from WT and ATG5-iKO CGNs in culture treated with BafA. (C–F) Axonal ER co-traffics with LC3-positive autophagosomes. (C) Kymographs showing colocalization and cotransport of DsRed-KDEL with GFP-LC3b-labeled autophagosomes in axons of WT hippocampal neurons. (D) The majority but not all of GFP-LC3b-labeled autophagosomes are positive for DsRed-KDEL. (E and F) Representative time series of colocalization and cotransport of DsRed-KDEL with GFP-LC3b-labeled autophagosomes in an axon. Shown in (E) is a representative time series of a proximal dendrite (F). Yellow arrows indicate moving DsRed-KDEL vesicles. (G) Hippocampal axons immunostained for endogenous LC3, Rtn3, and Tau. Blocking autophagy with a selective VPS34 inhibitor (1 μM, 24 h) and subsequent washout (4 h) results in axonal LC3 punctum formation positive for Rtn3 (i, example of a non-treated axon; ii, examples of VPS34 inhibitor-treated neurons after washout). (H–J) Acidification of neuronal ER in WT but not ATG5-iKO hippocampal neurons in culture. (H) Neurons transfected with EGFP-mCherry-RAMP4. EGFP is quenched as a result of low pH, causing a switch from GFP+/mCherry+ to GFP−/mCherry+ during lysosomal degradation of the ER. Yellow boxes indicate magnifications shown on the right. (I) GFP−/mCherry+ (acidified, red) RAMP4 is present in WT neurons but not in ATG5 KO neurons. Acidified ER is present again in ATG5-iKO neurons after co-expression with FLAG-ATG5. n = 6 experiments for WT and KO and n = 3 experiments for KO + FLAG-ATG5; total numbers of cells are indicated in bars. (J) ATG5 depletion does not influence acidification of mitochondria, measured by EGFP-mCherry-TOM20. n = 3 experiments; total numbers of cells are indicated in bars. See also Figure S4B. Scale bars, 5 μm. All data represent mean ± SEM. ^∗^p < 0.05.

We hypothesized that accumulation of axonal ER under conditions of ATG5 loss is a consequence of defective autophagy/lysosome-mediated turnover of tubular ER in axons, a process referred to as ER-phagy (Grumati et al., 2018; Khaminets et al., 2015; Liang et al., 2018). We first probed this by inhibiting lysosomal proteolysis by application of the v-ATPase inhibitor bafilomycin in astrocyte-free CGN cultures from WT or ATG5-iKO mice. Bafilomycin treatment of WT neurons for 24 h resulted in accumulation of ER membranes marked by Calnexin. In contrast, bafilomycin failed to cause a further elevation of Calnexin-positive ER membranes in ATG5-iKO neurons (Figures 5A and 5B), suggesting that ER accumulation in ATG5 KO neurons is indeed a result of defective autophagy/lysosome-mediated ER degradation. Calnexin also accumulated in astrocyte-free CGN cultures treated for 12 h with the autophagy inhibitor VPS34-IN1 (Figures S5A and S5B). Consistent with these biochemical data, we found ER tagged with DsRed-KDEL to efficiently co-traffic with LC3-EGFP-containing autophagosomes in distal axons of hippocampal neurons from WT (Figure 5E) but not from ATG5-iKO mice (Figures 5C and 5D). No co-transport of DsRed-KDEL-labeled ER membranes with LC3-EGFP-containing autophagosomes was observed in dendrites (Figure 5F). Furthermore, recruitment of endogenous LC3 to tubular ER membranes in the axon was observed upon acute pharmacological block and subsequent washout of VPS34-IN1 to reversibly induce neuronal autophagy (Figures 5G and S5B). These data indicate that the axonal ER is a prominent substrate of neuronal autophagy, eventually resulting in ER turnover in the neuronal soma, where most lysosomes reside. We directly tested this hypothesis using a recently developed biosensor for ER membrane turnover via autophagy (i.e., ER-phagy) (Liang et al., 2018). This sensor monitors lysosomal delivery of a chimeric reporter comprised of the pH-sensitive fluorescent protein EGFP (i.e., a probe quenched upon delivery to acidic lysosomes) and pH-insensitive mCherry fused to the ER membrane protein RAMP4. When expressed in WT hippocampal neurons, EGFP-mCherry-RAMP4 exhibited a reticular staining pattern, consistent with its ER localization, as well as distinctive mCherry-containing red fluorescent puncta corresponding to ER-containing acidic lysosomes. Such red fluorescent ER-containing acidic lysosomes were rarely observed in ATG5 KO neurons, consistent with a defect in ER-phagy caused by neuronal loss of ATG5. Defective ER-phagy was rescued by re-expression of ATG5 (Figures 5H and 5I). Surprisingly, loss of ATG5 did not affect autophagic turnover of mitochondria (i.e., mitophagy) (Figures 5J and S5C), consistent with data showing that ATG5 may be dispensable for mitophagy (Honda et al., 2014; Nishida et al., 2009). We conclude that accumulation of axonal ER under conditions of ATG5 loss is a direct consequence of impaired autophagy/lysosome-mediated turnover of tubular ER in axons.

### Elevated Calcium Release from ER Stores via RyRs Accumulated in Axons and at Presynaptic Sites Facilitates Neurotransmission in the Absence of ATG5-Mediated Neuronal Autophagy

**Figure 6:**
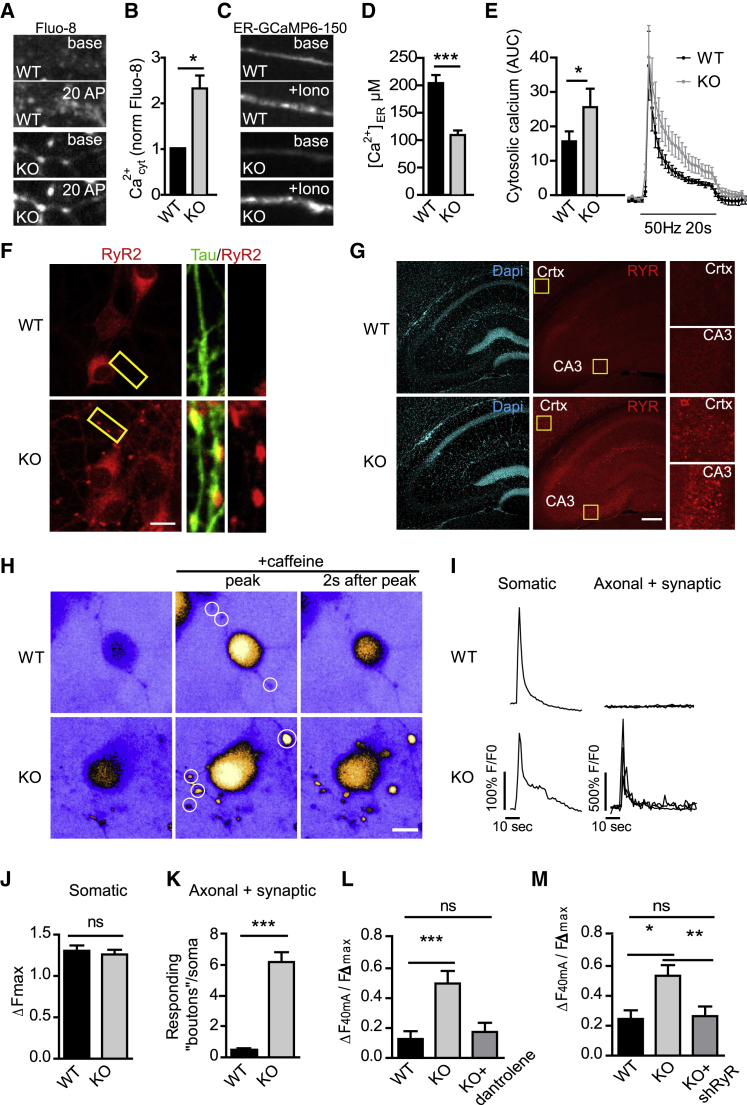
Increased RyR-Mediated Calcium Release Underlies Elevated Neurotransmission in ATG5 KO Neurons (A–E) Impaired calcium homeostasis in ATG5-iKO hippocampal neurons. (A and B) Hippocampal neuron cultures were probed with the fluorescent Ca^2+^-binding dye Fluo-8 to measure cytosolic calcium in neurites. Neurites were identified by a mild electrical stimulation (20 APs) causing a Fluo-8 increase. Fluorescence intensities of baseline Fluo-8 (before stimulus) are quantified in (B). Calcium levels in WT neurons were set to 1. n = 4 independent experiments, 36 images for WT and 38 images for KO; one-sample t test. (C) Hippocampal neuron cultures were transfected with ER-GCaMP6-150, and axons were imaged before and after 50 μM ionomycin application to induce indicator saturation for calibration. (D) Average peak fold change in fluorescence during ionomycin application is used to estimate resting ER calcium concentration in the axon. n = 30 axons, 3 independent experiments; unpaired t test. (E) Calcium buffering in the presynapse was measured by infecting neurons with the synaptophysin-GCaMP6 virus. The fluorescence change in response to a 50-Hz, 20-s pulse was measured. Average traces are indicated on the right, and the area under the curve (AUC) is plotted on the left. n = 5 independent experiments, 58–63 cells per condition; paired t test. (F) Hippocampal neuron culture immunostained for endogenous RyR2 and the axonal marker Tau. Scale bar, 5 μm. (G) Images of mouse brain sections showing an increase in RyR immunoreactivity in ATG5-cKO crtx and the hippocampal CA3 area. Yellow boxes indicate magnifications shown on the right. See also Figure S6A for quantifications. Scale bar, 200 μm. (H–K) Increased caffeine-induced calcium release from the ER in ATG5-iKO hippocampal neurons in culture. (H) Heatmap images showing Fluo-8 calcium responses during a 20-mM caffeine pulse. Scale bar, 10 μm. (I) Representative traces from Fluo-8 responses in the somata or axonal areas (indicated by white circles in H). (J) Maximum Fluo-8 intensity increase in WT and ATG5-iKO somata. n = 100 WT and 98 KO cells from two independent experiments; unpaired t test. (K) Average number of responding “boutons” per soma. n = 90 WT and 95 KO somata from two independent experiments; Mann-Whitney test. (L) Detection of exocytosis using Synaptophysin-pHluorin in WT and ATG5-iKO hippocampal neurons. A graph shows mean normalized peak fluorescence upon 40-mA stimulation. Dantrolene (10 μM), a RyR inhibitor, rescues increased responses in ATG5-iKO neurons. Values per cell are normalized to the corresponding maximal fluorescent peak at 100 mA (Fmax). n = 18–22 cells, 3 independent experiments; one-way ANOVA with Tukey’s post-test. (M) Ryanodine receptor (RyR) knockdown decreases exocytosis in ATG5-iKO neurons. Values per cell are normalized to the corresponding Fmax at 100 mA. n = 13 WT or 25–27 KO cells, 4 independent experiments; one-way ANOVA with Tukey’s post-test. All data represent mean ± SEM. ^∗^p < 0.05, ^∗∗^p < 0.01, ^∗∗∗^p < 0.001.

Major functions of the tubular ER are (1) transfer of phospholipids, such as phosphatidylinositol, across contact sites with the plasma membrane and (2) regulation of intracellular calcium signaling and homeostasis (Berner et al., 2018; Bezprozvanny and Kavalali, 2020; Saheki and De Camilli, 2017). We failed to detect significant alterations in the levels of phosphatidylinositol 4-phosphate and phosphatidylinositol 4,5-bisphosphate, the major products of plasma membrane lipid kinases that capitalize on substrate supply of phosphatidylinositol from ER membranes (Saheki and De Camilli, 2017), in ATG5 KO neurons (Figures S5D and S5E). Moreover, no change in the dynamics of axonal ER lumenal proteins were observed in fluorescence recovery after photobleaching (FRAP) experiments (Figure S5F) as might be expected if ER membrane integrity and function were compromised. Hence, we followed the alternative hypothesis that accumulation of tubular ER in axons might cause alterations in calcium homeostasis and facilitate calcium-triggered presynaptic neurotransmission (Bezprozvanny and Kavalali, 2020; Galante and Marty, 2003). We tested this hypothesis by assaying the relative calcium levels in the axoplasm of WT versus ATG5-iKO neurons using Fluo-8 as a reporter. Axoplasmic calcium levels were elevated about 2-fold in ATG5-iKO compared with WT neurons (Figures 6A and 6B). In contrast, quantitative measurement of the calcium concentration in the axonal lumen of the ER using ER-GCaMP6-150 (de Juan-Sanz et al., 2017) revealed a reduction from 200 μM in WT neurons to about 100 μM in ATG5-iKO neurons (Figures 6C and 6D). These data suggest that accumulation of tubular ER in axons of ATG5 KO neurons leads to elevated calcium efflux from the ER lumen into the axoplasm, which might conceivably disturb presynaptic calcium homeostasis. Indeed, when presynaptic calcium buffering in response to sustained train stimulation (50 Hz, 20 s) was probed by lentivirally encoded Synaptophysin-GCaMP6, we found a significantly reduced ability of ATG5 KO neurons to restore steady-state calcium levels (Figure 6E), suggesting a defect in calcium buffering, likely as a consequence of disturbed calcium homeostasis.

Defects in axonal and/ or ER calcium homeostasis might conceivably result from altered calcium entry via voltage-sensitive calcium channels (Cav), calcium efflux via the plasma membrane calcium ATPase (PMCA), influx into the ER via sarcoplasmic/endoplasmic reticulum calcium ATPase (SERCA), or elevated efflux from the ER lumen into the axoplasm via inositol 1,4,5-triphosphate receptors (IP_3_Rs) or RyRs (Del Prete et al., 2014; Jahn and Fasshauer, 2012; Nanou and Catterall, 2018; Neher and Sakaba, 2008; Scullin and Partridge, 2010). Quantitative proteomics and biochemical analysis by immunoblotting revealed a dramatic accumulation of RyRs (Figures 3G and S6B) in ATG5-iKO neurons and ATG5-cKO brains, whereas the levels of voltage-gated calcium P/Q channels (Cav2.1), PMCA, SERCA2, or various IP_3_R isoforms (IP_3_R1 and IP_3_R3) were unaltered (Figure 3G). Elevated steady-state levels of RyR in Tau-positive axons and in the forebrain were further confirmed by confocal imaging of ATG5-iKO hippocampal neurons (Figure 6F) and brain sections from ATG5-cKO mice (Figures 6G and S6A), respectively. Given the established function of RyRs in ER calcium homeostasis and in modulation of presynaptic neurotransmission (Galante and Marty, 2003; Irie and Trussell, 2017; Unni et al., 2004), we hypothesized that elevated calcium release from ER stores is mediated via RyRs accumulated in axons and at presynaptic sites to facilitate neurotransmission in the absence of ATG5. Consistent with this hypothesis, ATG5 KO neurons displayed a dramatic increase over WT controls in caffeine-induced calcium release via RyRs (Sato and Kamiya, 2011) in axons and presynapses (Figure S6C) but not in neuronal somata (Figures 6H–6K). Hence, axonal accumulation of RyRs causes RyR gain of function (i.e., facilitated release of calcium from axonal ER stores), consistent with our calcium imaging data (compare Figures 6A–6E). To finally determine whether increased calcium release from lumenal ER stores via RyR gain of function causally underlies elevated presynaptic neurotransmission, we targeted RyRs for acute pharmacological or sustained genetic perturbations. Pharmacological blockade of RyRs by dantrolene, a well-established RyR antagonist, or lentiviral knockdown of RyRs (Figure S6G) rescued elevated presynaptic neurotransmission in ATG5 KO neurons to amplitudes characteristic of WT neurons (Figures 6L, 6M, and S6D–S6F).

We conclude that elevated calcium release from ER stores via RyRs accumulated in axons and at presynaptic sites facilitates neurotransmission in the absence of ATG5-mediated neuronal autophagy.

## Discussion

Our collective data, based on cKO of ATG5 in excitatory neurons and quantitative proteomics as well as live imaging and electrophysiology, reveal a crucial function of neuronal autophagy in control of the tubular ER in axons to regulate excitatory neurotransmission via RyR-mediated calcium release from ER stores. This model is supported by several converging lines of evidence. First, we show that loss of neuronal autophagy in the absence of ATG5 facilitates excitatory neurotransmission in acute hippocampal slices (Figure 1) and in cultured hippocampal neurons (Figure 2) by increasing presynaptic release probability. Second, we identify, using SILAC-based quantitative proteomics analyses of nearly 2,000 neuronal proteins (Figure 3) combined with biochemical and optical imaging assays (Figure 5), components of the tubular ER (e.g., reticulons and the RyR) as the major substrates of neuronal autophagy. Strikingly, tubular ER accumulation was largely specific to axons and presynaptic sites (Figure 4) and was not observed in neuronal dendrites and only mildly (albeit insignificantly) in somata. The compartment specificity of ER accumulation in axons fits well with the observation that autophagosomes form primarily (although not exclusively) in distal axons and at presynaptic nerve terminals (Hill and Colón-Ramos, 2020; Maday and Holzbaur, 2014; Maday et al., 2012; Vijayan and Verstreken, 2017). Additional factors may contribute to the compartment-specific ER phenotype. For example, the peripheral tubular ER is closely linked to microtubule plus end-directed kinesin motors (Westrate et al., 2015; Zhang and Hu, 2016), likely resulting in effective retention of the tubular ER in axons that display a uniform plus-end-out microtubule polarity pattern. Third, we demonstrate that elevated calcium release from ER stores via RyRs accumulated in axons and at presynaptic sites of ATG5 KO neurons facilitates excitatory neurotransmission. These observations are consistent with recent data suggesting major roles of the ER (Bezprozvanny and Kavalali, 2020; de Juan-Sanz et al., 2017; Lindhout et al., 2019) and of calcium release via RyRs in control of presynaptic neurotransmission (Galante and Marty, 2003; Scullin and Partridge, 2010; Shimizu et al., 2008) and presynaptic forms of synaptic plasticity; e.g., long-term depression at hippocampal CA3-CA3 synapses (Unni et al., 2004). Facilitated RyR-mediated calcium release from axonal ER stores and concomitant elevation of glutamate exocytosis may explain neuronal cell death (Hernández et al., 2018; Wang and Qin, 2010; compare Figures S1D and S1E) and the strongly impaired postnatal viability of ATG5-cKO mice *in vivo*. The role of axonal ER-localized RyRs in calcium-triggered facilitation of presynaptic neurotransmitter release described here and before (Galante and Marty, 2003) appears to be distinct from the postulated function of STIM1, an ER protein known to couple to ORAI in the plasma membrane to mediate store-operated calcium entry (Saheki and De Camilli, 2017), in local regulation of release probability via a so far unknown mechanism (de Juan-Sanz et al., 2017).

In addition to their function in regulation of neurotransmitter release (Galante and Marty, 2003; Unni et al., 2004; this work), RyRs have been found to be located in close apposition to large conductance voltage-gated plasma membrane BK channels to rapidly regulate AP burst firing (Irie and Trussell, 2017). It is therefore possible that the observed accumulation of RyRs in the axonal ER of ATG5 KO neurons, in addition to its effects on presynaptic release probability and SV fusion, alters AP shape and, therefore, neuronal excitability. Consistent with this hypothesis, it has been found recently that loss of autophagy increases the excitability of striatal spiny projection neurons (Lieberman et al., 2020). Future experiments will need to test this possibility in detail.

Accumulation of the ER with associated neurodegeneration has been observed in CRISPR KO mice constitutively lacking the autophagy regulatory factor WDR45 (Wan et al., 2020). Our findings are consistent with this and further suggest that ER-phagy is a major autophagic process in neurons in the absence of proteotoxic challenges at steady state. The physiological and pathophysiological importance of ER-phagy in neurons is further underscored by the fact that loss FAM134B, an adaptor for selective autophagy of the reticular sheet ER, causes sensory neuropathy because of neurodegeneration in mutant mice and humans (Khaminets et al., 2015). A number of other adaptors for different forms of ER-phagy have been identified since then (Grumati et al., 2018). Whether any of these adaptor proteins are required for axonal ER-phagy in hippocampal neurons described here is unclear. Our own preliminary data (Figure S6H) argue against this. It is possible that the known ER-phagy adaptors are functionally redundant or that so far unidentified adaptors mediate autophagy of axonal ER-phagy in CNS neurons. Alternatively, axonal ER-phagy may be a constitutive process intimately linked to formation of autophagosomes in distal axons and at presynaptic sites (Hill and Colón-Ramos, 2020; Maday and Holzbaur, 2014; Maday et al., 2012; Vijayan and Verstreken, 2017) that serves a homeostatic role in coupling presynaptic function to constitutive turnover of RyR-containing axonal ER membranes.

In addition to the largely constitutive formation of autophagosomes in axons, autophagy has been shown to be induced by various conditions ranging from overexpression of aggregation-prone proteins (Corrochano et al., 2012) and ROS-induced protein oxidation (Hoffmann et al., 2019) to depletion of AZ proteins required for presynaptic function (Okerlund et al., 2017). Although we did not detect alterations in the steady-state levels or half-lives of major exo-endocytic and AZ proteins in ATG5 KO neurons (compare Figures 2 and 3; Table S1), our data are not incompatible with these earlier studies. For example, it has been shown that co-depletion of the giant AZ proteins Piccolo and Bassoon triggers activation of E3 ubiquitin ligases and key ATG proteins, resulting in targeting of SV proteins for degradation via the ubiquitin-proteasome system and autophagy (Waites et al., 2013), resulting in compromised synapse integrity. How different types of physiological (e.g., neuronal activity and aging) and pathophysiological stimuli (e.g., protein aggregate formation in neurodegenerative diseases) regulate distinct types of autophagy in different types of neurons (e.g., glutamatergic versus dopaminergic neurons) in the brain and in the peripheral nervous system remains a fruitful area for future studies.

## STAR★Methods

### Key Resources Table

**Table:** 

REAGENT OR RESOURCE	SOURCE	IDENTIFIER
**Antibodies**		

Active-Caspase3 (rabbit)	R&D Systems	Cat#AF835, RRID:AB_2243952
ATG5 (rabbit)	LifeSpan BioSciences Inc.	LS-C156610
Calnexin (rabbit)	Abcam	Cat#ab75801, RRID:AB_1310022
Calreticulin (rabbit)	Thermo Fisher Scientific	Cat# PA3-900, RRID:AB_325990
Cav2.1 (rabbit)	Synaptic Systems	Cat# 152 203, RRID:AB_2619841
CytochromeC (mouse)	BD Biosciences	Cat# 556433, RRID:AB_396417
DHRP (mouse)	Abcam	Cat# ab2864, RRID:AB_303365
FK2(ubi) (mouse)	Enzo Life Sciences	Cat# BML-PW8810 0500, RRID:AB_2051891
GAD65 (guinea pig)	Synaptic Systems	Cat# 198104, RRID:AB_10557995
GFAP (mouse)	Sigma-Aldrich	Cat# G3893, RRID:AB_477010
GluA1 (rabbit)	Millipore	Cat# ABN241, RRID:AB_2721164
GluN1 (mouse)	Synaptic Systems	Cat# 114 011, RRID:AB_887750
GM130 (mouse)	BD Biosciences	Cat# 610822, RRID:AB_10015242
Homer1 (rabbit)	Synaptic Systems	Cat# 160 003, RRID:AB_887730
HSC70 (rabbit)	Abcam	Cat# ab51052, RRID:AB_880538
IP3R1 (rabbit)	Cell Signaling Technology	Cat# 8568, RRID:AB_10890699
IP3R3 (rabbit)	Millipore	Cat# AB9076, RRID:AB_11212608
Kv pan (mouse)	UC Davis/NIH NeuroMab Facility	Cat# 75-392, RRID:AB_2491089
Kv1.1 (mouse)	UC Davis/NIH NeuroMab Facility	Cat# 75-007, RRID:AB_10673165
Kv1.2 (mouse)	UC Davis/NIH NeuroMab Facility	Cat# 75-008, RRID:AB_2296313
Kv1.4 (rabbit)	Novus Biologicals	NBP2-75552
Lamp1 (rat)	BD Biosciences	Cat# 553792, RRID:AB_2134499
LC3 (mouse)	MBL International	Cat# M152-3, RRID:AB_1279144
LC3B (rabbit)	Novus	Cat# NB600-1384, RRID:AB_669581
Map2 (mouse)	Sigma-Aldrich	Cat# M9942, RRID:AB_477256
Map2 (guinea pig)	Synaptic Systems	Cat# 188 004, RRID:AB_2138181
Munc13-1 (guinea pig)	Synaptic Systems	Cat# 126 104, RRID:AB_2619806
NaV pan (rabbit)	Gentex	GTX16935
P62 (guinea pig)	Progen	Cat# GP62-C, RRID:AB_2687531
PI(4)P (mouse)	Echelon Biosciences	at# Z-P004, RRID:AB_11127796
PI(4,5P2) (mouse)	Echelon Biosciences	Cat# Z-A045, RRID:AB_427211
Piccolo (rabbit)	Synaptic Systems	Cat# 142 002, RRID:AB_887759
PMCA (mouse)	Thermo Fisher Scientific	Cat# MA3-914, RRID:AB_2061566
PSD95 (guinea pig)	Synaptic Systems	Cat# 124 014, RRID:AB_2619800
Reticulocalbin 2 (rabbit)	Atlas Antibodies	Cat# HPA030694, RRID:AB_2673573
Reticulon 3 (rabbit)	Millipore	ABN 1723
RYR pan (mouse)	Enzo Life Sciences	Cat# ALX-804-016-R100, RRID:AB_2052517
RYR2 (guinea pig)	Frontier Institute	RyR2-GP-Af480
RyR2 (rabbit)	Millipore	Cat# AB9080, RRID:AB_11211327
Sec6b and anti-Sec61a (rabbit)	Tom Rapoport	N/A
Serca2 (mouse)	Thermo Fisher Scientific	Cat# MA3-919, RRID:AB_325502)
SNAP25 (mouse)	Synaptic Systems	Cat# 111 011, RRID:AB_887794
Sod2 (rabbit)	Abcam	at# ab13498, RRID:AB_300402
SV2A (mouse)	Pietro De Camilli	N/A
Synapsin (mouse)	Synaptic Systems	Cat# 106 001, RRID:AB_887805
Synaptobrevin 2 (guinea pig)	Synaptic Systems	Cat# 104 204, RRID:AB_2212601
Synaptotagmin1 (mouse)	Synaptic Systems	Cat# 105 011C5, RRID:AB_2619761
Synaptotagmin1 (luminal)-Cy3 (rabbit)	Synaptic Systems	Cat# 105 103C3 RRID:AB_887829
Tau1 (mouse)	Millipore	Cat# MAB3420, RRID:AB_11213630
Tubb3 (rabbit)	Synaptic Systems	Cat# 302 302, RRID:AB_10637424
Tubulin (mouse)	Sigma-Aldrich	Cat# T5168, RRID:AB_477579
VapB (rabbit)	Abnova Corporation	Cat# H00009217-D01, RRID:AB_10720965
VGAT (rabbit)	Synaptic Systems	Cat# 131 003, RRID:AB_887869
VGLUT (guinea pig)	Synaptic Systems	Cat# 135 304, RRID:AB_887878
Vinculin (mouse)	Sigma-Aldrich	Cat# V9264, RRID:AB_10603627
Goat anti mouse IgG Alexa Fluor 568	Thermo Fisher Scientific	Cat# A-11031 RRID: AB_144696
Goat anti mouse IgG Alexa Fluor 488	Thermo Fisher Scientific	Cat# A-11001; RRID: AB_2534069
Goat anti rabbit IgG Alexa Fluor 488	Thermo Fisher Scientific	Cat# A-11008; RRID: AB_143165
Goat anti rabbit IgG Alexa Fluor 568	Thermo Fisher Scientific	Cat# A-11011; RRID: AB_143157
Donkey anti rat IgG Alexa Fluor 488	Thermo Fisher Scientific	Cat# A-21208, RRID:AB_2535794
Goat anti guinea pig IgG Alexa Fluor 568	Thermo Fisher Scientific	Cat# A-11075, RRID:AB_2534119
Goat anti guinea pig IgG Alexa Fluor 647	Thermo Fisher Scientific	Cat# A-21450, RRID:AB_2735091
IRDye® 800CW Goat Anti-Mouse IgG	LI-COR Biosciences	Cat# 926-32210, RRID:AB_621842
IRDye® 680RD Goat anti-Mouse IgG	LI-COR Biosciences	Cat# 925-68070, RRID:AB_2651128
IRDye 680RD Goat anti-Rabbit IgG	LI-COR Biosciences	Cat# 926-68071, RRID:AB_10956166
IRDye® 800CW Goat Anti-Rabbit IgG,	LI-COR Biosciences	Cat# 926-32211, RRID:AB_621843
IRDye 680RD Donkey anti-Guinea pig IgG	LI-COR Biosciences	Cat# 926-68077, RRID:AB_10956079
IRDye® 800CW Donkey Anti- Guinea pig IgG	LI-COR Biosciences	Cat# 926-32411, RRID:AB_1850024

**Bacterial and Virus Strains**		

Lenti f(U6)sNLS-RFPw	Viral core facility of the Charité – Universitätsmedizin Berlin, Germany	Cat#BLV-360 https://vcf.charite.de/en/
Lenti f(syn)-Syp-GCamp6f-w	Viral core facility of the Charité – Universitätsmedizin Berlin, Germany	Cat#BLV-700 https://vcf.charite.de/en/

**Chemicals, Peptides, and Recombinant Proteins**		

Picrotoxin	Sigma-Aldrich	Cat#P1675
Tamoxifen ((Z)-4-Hydroxytamoxifen)	Sigma-Aldrich	Cat# H7904
Thapsigargin	Sigma-Aldrich	Cat# T9033
Dantrolene	Sigma-Aldrich	Cat# 251680
Ionomycin	Sigma-Aldrich	Cat# 407952
Bafilomycin A1	Enzo	Cat# BML-CM110-0100
Caffein	Sigma-Aldrich	Cat# c0750
Doxycycline hyclate	Sigma-Aldrich	Cat#D9891
D4-lysine	Silantes	Cat#211104113
13C6-arginine	Silantes	Cat#201204102
13C615N2-lysine	Silantes	Cat#211603902
13C615N4-arginine	Silantes	Cat#201603902

**Critical Commercial Assays**		

ProFection Mammalian Transfection System – Calcium Phosphate	Promega	Cat# E1200
Fluo-8H	AAT Bioquest	Cat# 21080

**Experimental Models: Organisms/Strains**		

Mouse: C57BL/6J	Charles River	RRID: IMSR_JAX:000664
Mouse: B6.129S-Atg5tm1Myok	RIKEN BioResource Center	Cat# RBRC02975, RRID:IMSR_RBRC02975
Mouse: BC.Cg-Tg(CAG-cre/Esr1^∗^)5Amc/J	The Jackson Laboratory	Cat# JAX:004682, RRID:IMSR_JAX:004682
Mouse: Emx1-Cre	RIKEN BioResource Center	Cat# RBRC01342, RRID:IMSR_RBRC01342

**Oligonucleotides**		

See Table S2		N/A

**Recombinant DNA**		

Synaptophysin -pHluorin	L. Lagnado	N/A
sRed2-Mito-7	Michael Davidson	RRID:Addgene_55838
TetOn-eGFP-mCherry-RAMP4	Liang et al., 2018	RRID:Addgene_109014
TOM20MTS-mCherry-EGFP-Tet-On	Liang et al., 2018	RRID:Addgene_09016
pEGFP-LC3	Lee et al., 2008	RRID:Addgene_24920
mRFP-LC3	Kimura et al., 2007	RRID:Addgene_ 21075
ER-GCAMP6-150	de Juan-Sanz et al., 2017	RRID:Addgene_ 86918
GCamp6f	Chen et al., 2013	RRID:Addgene_40755

**Software and Algorithms**		

Prism 5	Graph Pad	RRID: SCR_002798
Fiji (ImageJ)	NIH	RRID: SCR_002285
MaxQuant software	MaxQuant	RRID:SCR_014485
GOrilla: Gene Ontology Enrichment Analysis and Visualization Tool	Eden et al., 2009	RRID:SCR_006848
Etomo/IMOD	Kremer et al., 1996	https://bio3d.colorado.edu/imod/
Microscopy imaging browser MIB	Belevich et al., 2016	http://mib.helsinki.fi/index.html
BLOCK-iT RNAi Designer	Thermo Fisher Scientific	RRID:SCR_002794
Biosettia shRNA design	Biosettia	N/A
Image Studio Lite	LI-COR Biosciences	RRID:SCR_013715
SigmaPlot	Systat Software, Inc.	RRID:SCR_003210
IGOR Pro	WaveMetrics, Lake Oswego, OR	RRID:SCR_000325
PatchMaster software	Heka Elektronics	RRID:SCR_000034

### Resource Availability

#### Lead Contact

Further information and requests for resources and reagents should be directed to and will be fulfilled by the Lead Contact, Volker Haucke (haucke@fmp-berlin.de).

#### Materials Availability

All unique reagents (e.g., plasmids) generated in this study are available from the Lead Contact without restriction.

#### Data and Code Availability

Proteomics datasets related to Figure 3 in the paper are available in Table S1. Complete proteomics source data are available from the corresponding author on request. No further unique datasets or codes were generated in this study.

### Experimental Model and Subject Details

#### Animals

All animal experiments were reviewed and approved by the ethics committee of the “Landesamt für Gesundheit und Soziales” (LAGeSo) Berlin) and were conducted accordingly to the committee’s guidelines.

• Health/immune status: The animals have a normal health and immune status. The animal facility where the mice are kept is regularly checked for standard pathogens. The health reports can be provided upon request.
• Mice used for all experiments were naive. No drug tests were done. Mice were housed under 12/12-h light/dark cycle and up to five animals per cage, with access to food and water *ad libitum.*
• Mouse strains and crossings: ATG5f^lox/flox^ (B6.129S-Atg5tm1Myok) mice (Hara et al., 2006) were crossed with a tamoxifen inducible Cre line (Hayashi and McMahon, 2002) to generate ATG5-iKO (ATG^flox/flox^ × CAG-Cre). To delete ATG5 in excitatory neurons in neocortex and hippocampus, ATG5^flox/flox^ mice were crossed with an Emx1-Cre line (Iwasato et al., 2000) generating ATG5^flox/-^ × EMX1-Cre mice (first generation). By mating ATG5^flox/-^ × EMX1-Cre with ATG5^flox/flox^ mice we obtained conditional ATG5^flox/flox^ × EMX1-Cre (ATG5-cKO) mice.
• Sample size estimation: No estimation of simple size was done as sample sizes were not chosen based on pre-specified effect size. Instead, multiple independent experiments were carried out using several biological replicates specified in the legends to figures.
• Age and gender of subjects or animals: Mice from both genders were used for experiments. Electrophysiological experiments were conducted using 2-3 months-old ATG5lox/lox × EMX1-Cre and corresponding control mice. Neuronal cultures were prepared from postnatal mice at p1-3 (hippocampus) or p4-7 (cerebellum). Immunohistochemistry or immunoblotting was conducted by analyzing 2-5 months-old ATG5 KO mice and their WT littermates.
• How subjects/samples were allocated to experimental groups: Littermates were randomly assigned to experimental groups. Multiple independent experiments were carried out using several biological replicates specified in the figure legends.

### Method Details

#### Electrophysiology

##### Slice preparation and instrumentation

Electrophysiology was performed in slices prepared from 2-3 months-old ATG5lox/lox × EMX1-Cre and corresponding control mice. Slices were prepared in oxygenated (95% O_2_ / 5% CO_2_) dissection artificial cerebrospinal fluid (ACSF) at low temperature (3-4°C) using vibroslicer (Leica, VT 1200S). After preparation slices were recovered in a resting chamber (Harvard apparatus, BSC-PC) containing ACSF at room temperature (22-24°C) for at least 1.5 hour before recordings. Recordings were performed in a chamber (Warner instruments RC-27L) filled with ACSF with a solution exchange of 3-5 mL per min at room temperature. An upright microscope (Olympus, BX61WI) was used for slice positioning and electrode placement. Glass stimulating (1-1.5 MΩ) and recordings (1.5-2.5 MΩ) electrodes filled with ACSF were prepared from glass capillaries (Hilgenberg) using micropipette puller Sutter P-1000 (Sutter Instruments). The data were recorded at a sampling rate of 10 kHz, low-pass filtered at 3 kHz using EPC9 amplifier and analyzed using Patch Master software (Heka Elektronics).

##### Recordings of CA1 fEPSPs

Mice decapitated after cervical dislocation and brain quickly extracted into dissection ACSF containing: 2.5 mM KCl, 1.25 mM NaH_2_PO_4_, 24 mM NaHCO_3_, 1.5 mM MgS0_4_, 2 mM CaCl_2_, 25 mM glucose, 250 mM sucrose (pH 7.35-7.40). 350 μm thick transversal slices containing clearly visible hippocampus were prepared from both hemispheres and collected in a resting chamber filled with resting/ recording ACSF supplemented with 120 mM NaCl instead of 250 mM sucrose. After recovery slices transferred into recording chamber stimulation and recording electrodes placed in a visually preselected area of *stratum radiatum* and slowly advanced until maximum responses were obtained. Electrical stimuli of 0.2 ms duration were delivered at 0.05 Hz at the stimulation intensity which induced approximately 30%–50% of the maximum responses as baseline stimuli. After stabile baseline recordings of at least 10 min an input/output stimulus response curves were made as a measure of basal excitatory synaptic transmission. Slopes of the fEPSP were plotted against fiber volley (FV) amplitudes as a function of increasing stimulation intensity. Stimulation intensity was increased until the maximal fEPSP were obtained, defined as a response with superimposed population spike (PS) component on decaying fEPSP responses. In experiments performed with presence of GABAR antagonist Picrotoxin (50 μM), to prevent spontaneous epileptiform activity, we introduced a cut with a sharp blade between CA3 and CA1 regions. Short-term synaptic facilitation was tested by delivering two pulses at time intervals from 10 to 500 ms at a stimulation intensity which induced one third of the maximal responses. Paired pulse facilitation (PPF) was calculated as a percentage increase of the slope of the second response as compared to the first. For short intervals (10 and 20 ms), the first fEPSPs were digitally subtracted before measurements of the second fEPSPs. Each trace measured for the stimulus response curve and paired pulse parameters is an average of 3 consecutive stimulations delivered every 20 and 30 s for stimulus response curves and paired pulse protocols, respectively.

NMDA receptor-mediated fEPSPs were isolated to estimate release probability using the use-dependent irreversible NMDA receptor antagonist MK-801. Stimulation intensity was set to 60%–70% of the maximum responses and stimulated every 20 s in the presence of AMPA/kainite receptor antagonist NBQX (10μM) and GABA_A_/glycine receptor antagonist Picrotoxin (50μM). Initial AMPA receptor-mediated responses were taken as 100%. We reduced extracellular Mg^2+^ ion concentration from 1.5 to 0.25 mM in order to uncover NMDA receptor- mediated responses. Stabile NMDA receptor-mediated responses were isolated for 40-50 min. The amplitudes of AMPA and NMDA receptor responses were measured in their maximal peak area and plotted as NMDA/AMPA ratios. The non-competitive open channel NMDA receptor antagonist MK-801 (30μM) was applied for 10 min before and 30 min during stimulation to measure the decay kinetics of NMDA receptor-mediated responses. At the end of every experiment the potent NMDA receptor antagonist APV (50μM) was applied. APV reduced the responses further to about 2% of the initial value. To calculate the decay of NMDA receptor-mediated responses, the first response was taken as 100% and a mono exponential decay curve was applied for each individual experiment to allow the determination of τ values.

##### Recordings of MF-fEPSPs

Mice anesthetized with isoflurane and transcardially perfused with ice cold dissection ACSF containing the following substances: 75 mM sucrose, 25 mM glucose, 87 mM NaCl, 25 mM NaHCO_3_, 2.5 mM KCl, 1.25 mM NaH_2_PO_4_, 0.5 mM CaCl_2_, 7 mM MgCl_2_, pH 7.35-7.4. Dissection ACSF was cooled down in a freezer and bubbled at least 30 min prior to use with 95% O_2_ / 5% CO_2_. After 2 minutes of perfusion brain quickly removed and fresh 350 μm-thick hippocampal sections were prepared from both hemispheres and kept in sucrose based cutting/storage solution for recovery at 35°C for 30 minutes as described in Bischofberger et al. (2006). Slices were transferred in a resting chamber filled with recording ACSF of following composition: 120 mM NaCl, 2.5 mM KCl, 1.25 mM NaH_2_PO_4_, 25 mM NaHCO_3_, 1.5 mM MgS0_4_, 2.5 mM CaCl_2_, 25 mM glucose, pH7.35-7.4, at room temperature for at least an hour before the use. Mossy fibers (MF) were stimulated in the area of internal side of granule cell layer of the dentate gyrus and MF-*fEPSPs* were recorded in the str. lucidum of the CA3 field. MF-CA3 responses are characterized with the strong presynaptic facilitation and were identified using frequency facilitation parameter in which stimulation frequency is set to 0.3 Hz. The responses which exhibit at least 200% facilitation were accepted as MF-fEPSPs and were recorded further. Basal stimulation was applied every 30 s in order to monitor stability of the responses at least for 15 minutes before LTP recordings. The stimulation intensity for FF and LTP experiments were selected to 50%–60% and 5 HFS delivered every 30 s each one containing 100 pulses at 100Hz were applied to induce LTP. LTP at this synapse can be generated presynaptically and is known to be NMDA receptor-independent, therefore 50 μM APV was bath applied during recordings. In order to confirm that fEPSPs were generated by the stimulation of MFs an agonist of type II metabotropic glutamate receptors DCG IV (2 μM) was applied and only responses inhibited by 70%–80% and more were assumed to be elicited by mossy fiber synapses.

##### Whole cell recordings

Slices were recorded in a submerged recording chamber and were perfused with ACSF at a flow rate of 5 ml/min. Whole-cell recordings were performed with a K-gluconate–based intracellular solution containing (in mM) K-gluconate (120), HEPES 20, KCl 3, NaCl 7, MgATP 4, NaGTP (0.3), and phosphocreatine 14, adjusted to pH 7.3 with KOH. Gabacine (1μM) and APV (50μM) were added to the ACSF to block GABA-ergic transmission and to prevent epileptic activity or LTP induction, respectively. Paired pulse ratio (PPR) was detected by Schaffer collateral stimulation with a low resistance glass electrode in str. radiatum of CA1. Paired stimulation (50 ms ISI) was applied and the amplitude of the second EPSC was devided by the first EPSC amplitude. Cumulative distribution of PPR was analyzed using 10 PPRs per cell.

Spontaneous EPSCs (sEPSCs) were recorded in voltage clamp configuration and cells were clamped to −60mV. Signals were detected automatically using IGOR Pro with the plugin Neuromatics and subsequently manually sorted by visual inspection. Cumulative distribution of sEPSC interevent interval (IEI) was analyzed using an equal number of events per cell per condition to prevent overrepresentation of single neurons. Only cells where at least 30 IEIs could be detected were taken into account for the distribution.

Release probability was detected using a minimal stimulation protocol in 30-60 traces by detecting the number of traces in which stimulation induced or failed to induce an EPSC. To determine the correct stimulation intensity for minimal stimulation, we used a paired pulse (50 ms ISI) protocol. The release probability thus refers to the release probability of the recorded synaptic connection. For detection of the readily releasable pool (RRP), the stimulation intensity was set to induce an EPSC with 50% of the maximal amplitude. 500 pulses were applied with 20Hz to result in a replenishment of synaptic vesicles. EPSC amplitudes were cumulatively plotted and the slope of the last 50 values was extrapolated and the intercept with the y axis represents the RRP size (Kaeser and Regehr, 2017; Schneggenburger et al., 1999).

#### Expression constructs, shRNA and lentivirus production

Synaptophysin 1 fused to pHluorin was kindly provided by L. Lagnado (MRC Laboratory of Molecular Biology, Cambridge, UK). ER-GCAMP6-150, TetOn-eGFP-mCherry-RAMP4, TOM20MTS-mCherry-EGFP-Tet-On, sRed2-Mito-7 and pEGFP-LC3 were obtained from Addgene. DsRed-KDEL was created by inserting an ER retention signal sequence (AAGGACGAGCTG) in a pDsRed2 expression vector just before the stopcodon.

For viral-mediated expression, lentiviral vectors expressing synaptophysin fused C-terminally with GCamp6f controlled by the human synapsin-1 promotor, were used. For viral-mediated knockdown, lentiviral vectors expressing nuclear localized RFP controlled by the human synapsin-1 promotor, and the appropriate shRNA controlled by the U6 promoter, were used. For target and non-target control shRNA sequences see Table S2. Lentiviral particles were produced by the viral core facility of the Charité – Universitätsmedizin Berlin, Germany. See Key Resources Table for further information.

#### Antibodies

See Key Resources Table.

#### Neuron preparation, culture, infection, and transfection

Neuronal cultures were prepared by surgically removing the hippocampi or cerebellum from postnatal mice at p1-3 (hippocampus) or p4-7 (cerebellum), followed by trypsin digestion to dissociate individual neurons. 100,000 hippocampal cells were plated as 40 μL drops per poly-L-lysine coated coverslip and 2 mL of plating medium (basic medium (MEM; 0.5% glucose; 0.02% NaHCO3; 0.01% transferrin) containing 10% FBS, 2 mM L-glutamine, insulin and penicillin/streptomycin) was added 1 h after plating. For cerebellar granule cell (CGN) cultures 1.5x106 cells were added directly to poly-L-lysine coated dished containing 2 mL of plating medium. After one day *in vitro* (DIV1) 1 mL of plating medium was replaced by 1 mL of growth medium (basic medium containing 5% FBS; 0.5 mM L-glutamine; 2% B27 supplement; penicillin/ streptomycin) and on DIV2 1 mL of growth medium was added. AraC was added to the culture medium to limit glial proliferation. For cerebellar granule cell (CGN) cultures 25mM KCl was added to the plating and growth medium. CGN cultures used for the multiplexed SILAC are grown in Neurobasal medium (described in more detail under the Multiplexed SILAC subheading). To initiate homologous recombination in neurons from floxed animals expressing a tamoxifen-inducible Cre recombinase cultured neurons were treated with 0.3 μM (Z)-4-hydroxytamoxifen (Sigma) immediately after plating. When other drugs are added to the growth medium, concentration and duration of treatment are mentioned in the figure legends.

For lentiviral transduction about 5x10^5^ infectious virus units per 35 mm-diameter well were pipetted onto hippocampal neurons at DIV 1 or 2. A non-targeting shRNA control was included in RYR knockdown experiments. For calcium phosphate transfection 6 μg plasmid DNA, 250 mM CaCl_2_ and water (for each well of a 6-well plate) were mixed with equal volume of 2x HEPES buffered saline (100 μl) and incubated for 20 min allowing for precipitate formation, while neurons were starved in NBA medium for the same time at 37°C, 5% CO2. Precipitates were added to neurons and incubated at 37°C, 5% CO2 for 30 min. Finally, neurons were washed three times with HBSS medium and transferred back into their conditioned medium. For TetOn-eGFP-mCherry-RAMP4/TOM20 expression, 4 μg/ml doxycycline was added at the day of transfection. Live imaging and fixation of hippocampal cultures was conducted at DIV 13–16, CGN cultures were lysed at DIV13-20.

#### Immunostaining of hippocampal neurons in culture

Neurons were fixed on DIV 13–16 with 4% paraformaldehyde (PFA)/4% sucrose in phosphate-buffered saline (PBS) for 15 min at room temperature (RT), washed and incubated with primary antibodies in PBS containing 10% normal goat serum (NGS) and 0.3% Triton X-100 (Tx) overnight at 4 degrees. Coverslips were washed three times with PBS (10 min each) and incubated with corresponding secondary antibodies for 1 hour. Finally, coverslips were washed three times in PBS and mounted in Immumount. Alternatively, for LC3 immunostaining, cells were fixed with PFA and permeabilized with digitonin (200 μg/ml) for 15 min before incubating with primary and secondary antibodies in PBS. For lipid stainings, cells were fixed with 2% PFA/2% sucrose/ 1% glutaraldehyde in PBS for 20 min at RT. Neurons were then permeabilised with 0.5% Saponin /1% BSA in PBS for 30 min at RT and incubated with indicated antibodies diluted in 1%BSA/10%NGS in PBS. For live labeling of synapses, neurons were incubated with Synaptotagmin-Cy3 for 10 minutes in conditioned medium at 37°C, washed three times and prepared for calcium imaging (see below). Fixed neurons were imaged at a resolution of 1,024 × 1,024 on a Zeiss laser scanning confocal microscope LSM710 or a spinning disc confocal microscope (CSU-X1, Nikon) with a 63 × oil objective. All acquisition settings were set equally for all groups within each immunostaining. Image processing and quantitative analysis was performed in ImageJ. For quantitative analysis of fluorescent intensities in the soma the total area of the soma was manually selected and measured using ImageJ selection tools. Average intensities of fluorescent puncta (synapses) were measured by centering 9 × 9 pixel (~1 × 1 μm) regions on maxima determined by ImageJ processing function. SynapCountJ, an ImageJ plugin, was utilized to determine synapses via colocalization of Homer and vGLUT in traced neurites, as described previously (Mata et al., 2016). For quantifying ER antibody stainings in neurites MAP2 and Tau signal were used as template for a mask, restricting the quantified area to the shape of the dendrites or axons. For quantifiying lipid levels in axons, Synaptobrevin staining signals were used as a mask. Fluorescent areas were determined by applying thresholding and analyzed using the ‘Analyze particles’ ImageJ module to determine the number or area of fluorescent spots.

#### Immunohistochemistry on brain sections

2-5 months-old ATG5 KO mice and their WT littermates were euthanized by an overdose (i.p.) of Ketamin (120 mg/kg body weight)/Rompun (16 mg/kg body weight) and transcardially perfused with 4% formaldehyde in 0.1 M PBS. Brains were isolated and postfixed in the same solution overnight at 4°C. After cryoprotection in 20% sucrose, frozen sections (30 μm) were collected in 0.1 M PBS. For immunostaining, corresponding hippocampal sections from WT and KO littermates were processed simultaneously. Sections were blocked for 2 h in 5% normal goat serum and 0.125M PBS with 0.3% Tween (PBST). Tissue was then washed with PBST and incubated in normal goat serum–PBST mixture for 48 h with primary antibodies. After washing, sections were incubated for 16 h with Alexa-conjugated secondary antibodies and Dapi in PBST. Finally, sections were washed, mounted and coverslipped on gelatin-coated glass slides. Sections were imaged at a resolution of 1,024 × 1,024 using a Zeiss laser scanning confocal microscope LSM710 with a 20x (dry) or 40x (oil) objective. All acquisition settings were set equally for sections of all groups within each immunostaining. Image processing and quantitative analysis of fluorescence intensity was performed in ImageJ. Images were quantified by measuring the mean intensity in defined region of interests (ROI). To quantify RYR area images were thresholded and particles analyzed with the analyze function within defined ROIs. Only particles with sizes larger than 4 pixels were selected for analysis. For synapse count in CA1 areas, 6 ROIs of 20x20 μm were analyzed per animal. Homer1 immunostaining was used as a mask to count synaptic vGLUT particles using the particle analyzer function in ImageJ.

#### pHluorin imaging

To track synaptic vesicle exo-/ endocytosis, neurons transfected with synaptophysin-pHluorin were subjected to electrical field stimulation using an RC-47FSLP stimulation chamber (Warner Instruments) and imaged at 37°C in imaging buffer (170 mM NaCl, 3.5 mM KCl, 0.4 mM KH_2_PO_4_, 20 mM N-Tris[hydroxyl-methyl]-methyl-2-aminoethane-sulphonic acid (TES), 5 mM NaHCO_3_, 5 mM glucose, 1.2 mM Na_2_SO_4_, 1.2 mM MgCl_2_, 1.3 mM CaCl_2_ (unless stated otherwise), 10 μM CNQX and 50 μM AP-5, pH 7.4) by epifluorescence microscopes (Zeiss Axiovert 200M or Nikon Eclipse Ti) equipped with a 40Χ oil objective. Images were acquired at 0.5 or 1 Hz frame rate. Quantitative analysis of responding boutons (20 per stimulation) was performed using ImageJ. Fluorescence intensities of responding boutons were corrected for background and photobleaching, if nessesary. For the experiments in which stimulation intensities were varied, each cell was subjected to the different stimulation strengths mentioned (e.g., 20mA-30mA-40mA-50mA-100mA). For the experiments in which calcium concentrations were varied, the stimulation strength was fixed at 100mA and each cell was subjected to the different calcium concentrations mentioned. ΔF was obtained by calculating ΔF = [F (data point fluorescence) - F0 (resting fluorescence)]. ΔFmax is ΔF during a 100mA stimulation or 0.8mM calcium.

#### Photobleaching experiments

For quantitative fluorescence recovery after photobleaching (FRAP) experiments, neurons were transfected as described before, and imaged on a Zeiss laser scanning confocal microscope LSM710 with ZEN 2010 software. The acquisition was performed with a 63X oil objective, 1024 × 1024 pixels per image and a zoom factor 4.5. After acquiring 10 pre-FRAP images (every 5 s), an 80 pixel long ROI on the proximal axon was photobleached with maximal laser power (10 iterations) and a further 30 images were acquired. To analyze the recovery of fluorescence, the bleached area was selected and background subtracted by subtracting the intensity of an empty, non-bleached area. Recovery R was calculated as R = (I(t)-I(directly after bleaching))/(I(before bleaching)-I(directly after bleaching)), with I denoting total intensity.

#### Ca^2+^ imaging

##### Cytosolic Ca^2+^:

Hippocampal neuron cultures from WT and ATG5 KO mice were loaded with 2 μM Fluo-8/AM together with 0.02% pluronic for 15 min at 37°C. Prior to imaging, neurons were washed 3 times in imaging buffer (see heading pHluorin Imaging for recipe). For data shown in Figures 6A and 6B neurites were identified by a mild 20AP electrical stimulation using a RC-47FSLP stimulation chamber (Warner Instruments) causing a Fluo-8 increase. For the caffeine-induced calcium responses (Figure 6H), calcium was omitted from the imaging buffer and images were acquired at 1 Hz frame rate. After correction for background fluorescence, fluorescence intensity was analyzed. Number of responding boutons per soma (Figure 6K) was determined by counting the responding boutons in a 100x100 μm ROI containing a soma.

##### Synaptic Ca^2+^:

Neurons were transduced with Synaptophysin-GCamp6 as described before and subjected to electrical field stimulation using an RC-47FSLP stimulation chamber (Warner Instruments) and imaged in imaging buffer. Images were acquired at 1 Hz frame rate.

##### ER luminal Ca^2+^ measurements:

Neurons were transfected with ER-GCAMP6-150 as described before, and axons were imaged in imaging buffer before and after (Fmax) addition of 50 μM ionomycin. Knowing the *in vitro* characteristics of the indicator used (de Juan-Sanz et al., 2017), baseline [Ca^2+^]ER is calculated using the following equation:

$$\left[Ca^{2+}\right]ER=Kd(\left(Fr/Fmax-1/Rf\right)/{\left(1-Fr/Fmax\right)}^{1/n}$$

Kd is the affinity constant of the indicator (150 μM), Fr is the measured fluorescence at rest, Rf is the dynamic range (45) and n is the Hill coefficient (1.6). Fmax values were not corrected for pH changes.

All Ca^2+^ imaging experiments were performed in imaging buffer at 37°C with an epifluorescence microscope (Nikon Eclipse Ti) equipped with a 40Χ oil objective. Quantitative analysis and image processing were performed using ImageJ.

#### Electron microscopy and tomography

DIV14 neurons were fixed with 2% glutaraldehyde in PBS. Coverslips were then postfixed with 1% OsO_4_ and 1.5% potassium hexacyanoferrat (III), stained en bloc with 1% uranyl acetate, followed by dehydration in a methanol gradient, propylene oxide and Epoxy resin infiltration. After polymerization, coverslips were removed and 50 nm sections were cut and contrasted with uranyl acetate and lead citrate for transmission electron microscopy (TEM) and morphometric analysis (SVs). For TEM tomography, 250 nm sections were cut and collected on coated slotted grids with 10 nm gold fiducials. Series of images from +60° to −60° were taken with a 1° step at Tecnai G20 microscope. Etomo/IMOD and Microscopy imaging browser MIB were used to work with 3D volumes and render 3D models of subsynaptic structures. For the determination of synapse number and density in the CA1 area, 300 μm slices of 2% glutaraldehyde and 4% PFA PBS-perfused brains were postfixed with 1% OsO_4_ and processed for Epoxy embedding similar to the analysis of cultured neurons. Following resin polymerization, semithin sections were used to localize the proximal part of the CA1 *stratum radiatum* for ultrathin sectioning. Gross morphological assessments were performed blindly of genotype. Samples were also analyzed for the presence or absence of apoptotic or necrotic cells or neurites, neurite free areas in the CA1 neuropil (“holes”) and other potential abnormalities. No signs of tissue necrosis were observed. The density of synaptic profiles per CA1 neuropil area was assessed by counting clearly recognizable postsynaptic elements (spine heads with postsynaptic density) in large CA1 *stratum radiatum* neuropil overviews.

#### Immunoblot analysis of mouse brain extracts and neuron cultures

Brain tissue was homogenized in lysis buffer (20 mM HEPES-KOH, pH 7.4, 100 mM KCl, 2 mM MgCl2, 1% Triton X-100, supplemented with 1 mM PMSF and mammalian protease and phosphatase inhibitor mixture) using a glass teflon homogenizer. Neuron cultures were lysed in RIPA buffer (150 mM NaCl, 1.0% NP-40, 0.5% sodium deoxycholate, 0.1% SDS, 50 mM Tris, pH 8.0) with protease and phosphatase inhibitors. Lysates were incubated 30 min on ice before centrifugation at 17,000 g for 10 min at 4 °C and protein concentrations determined by Bradford or BCA assay. Equal concentration of lysates in Laemmli sample buffer were boiled for 5 min. Between 20 and 60 μg protein was resolved by SDS–PAGE and immunoblotting was done on nitrocellulose membranes. Membranes were incubated with the primary antibodies at 4°C overnight. On the next day, bound primary antibodies were detected by incubation with IRDye 680/800CW-conjugated secondary antibodies via the Odyssey Fc Imaging system (LI-COR Biosciences).

#### Multiplexed SILAC and mass spectrometry analysis

CGN WT and KO cultures (1.5-1.7x106 cells per culture) were grown in custom-made lysine and arginine-free NB (Life technologies) to which “medium” (M) variants D4-lysine/13C6-arginine (Lys4/Arg6) or “heavy” (H) variants 13C615N2-lysine/13C615N4-arginine (Lys8/Arg10) were added. Growth medium consisted of (Lys/Arg) NB medium supplemented with 2% B-27, 0.5 mM L-glutamine, 25mM KCL and penicillin/streptomycin. After 2 weeks, the cultures were gently washed and growth medium was replaced by conditioned medium from “sister cultures” grown in paralel in “light” (unlabeled Lys/Arg) growth medium. Neurons were harvested and lysed after 0, 2 and 6 days and mixed together as pairs of time-matched WT and KO sets. To exclude the possibility of a specific labeling type affecting the experimental outcome, the labeling (heavy or medium type) was varied between the WT and KO samples in the four biological replicates. For clarifying purposes in text, figures and legends, the KO is always heavy labeled (H) and the WT is medium labeled (M).

Forty micrograms of protein in Laemmli sample buffer from each time point was separated on 4%–15% SDS–PAGE, each lane was then cut into 15 slices, and in-gel tryptic digestion was performed. Tryptic peptides were analyzed by a reversed-phase capillary liquid chromatography system (Ultimate 3000 nanoLC system; Thermo Scientific) connected to an Orbitrap Elite mass spectrometer (Thermo Scientific). Identification and quantification of proteins were performed using MaxQuant (version 1.5.1.0) software. Data were searched against the Uniprot mouse protein database. The initial maximum mass deviation of the precursor ions was set at 20 ppm, and the maximum mass deviation of the fragment ions was set at 0.35 Da. Methionine oxidation and the acrylamide modification of cysteine were used as variable modifications. False discovery rates were < 1% based on matches to reversed sequences in the concatenated target-decoy database. Proteins were considered if at least two sequenced peptides were identified.

#### Data analysis of SILAC

SILAC quantitation is done using the signals of the medium (Lys4/Arg6) and heavy (Lys8/Arg10) labeled peptides, the unlabeled peptides are ignored. Four independent experiments were performed to compare protein degradation in WT versus KO cultures after 6 days of “light” medium. Only proteins with a H/M ratio in both time points (t0 and t6) in 3 out of 4 experiments were considered. The plotted fold changes were calculated by dividing H/M(t6) by H/M(t0). Analyses were performed using Microsoft Excel. Synaptic proteins were manually selected using a list of 314 proteins that are either synapse-specific, highly enriched or implicated in synaptic function (Hakim et al., 2016). GO cellular component enrichments were calculated using GOrilla, using a ranked list of proteins with > 1.5-fold change (> 0.6 log_2_fold) in KO/WT ratio and the total list of 1753 proteins as a reference. The GO subcellular localization of the 73 hit proteins (defined as > 0.6 log_2_-fold change and p < 0.05) was done manually for each hit using the UniProtGO Annotation Database.

#### Experimental Design

A strategy for randomization, stratification or blind selection of samples has not been carried out. Sample sizes were not chosen based on pre-specified effect size. Instead, multiple independent experiments were carried out using several sample replicates as detailed in the figure legends.

### Quantification and Statistical Analysis

#### Imaging and biochemistry

Values are always depicted as mean ± SEM. Significance is denoted using asterisks ^∗^p < 0.05, ^∗∗^ p < 0.01, ^∗∗∗^ < 0.001 and p > 0.05 is not significant (ns). Statistical data evaluation was performed using Graph Pad Prism 5 software. One-sample t tests were used for comparisons with control group values that had been set to 1 for normalization purposes. For comparisons between two experimental groups statistical significance was analyzed by two-sample, two-tailed unpaired or paired Student’s t tests or Mann–Whitney test (as indicated in the figure legends). Pearson’s chi-square test was used to examine Mendelian ratios. Kolmogorov–Smirnov test was performed to compare the distributions of individual genotypes for data shown as cumulative distribution. For comparisons between more than two experimental groups statistical significance data was analyzed by one-way ANOVA with post hoc test (as indicated in the figure legends). The number of animals, cell cultures or cells used (n) is stated in the figure legends. SigmaPlot was used for electrophysiological data analyses, presentation and statistical calculations. Data curves were statistically evaluated using ANOVA with repeated-measures (significance depicted over a line encompassing the curve) and comparisons of two groups statistical significance was tested using a two-tailed unpaired Studentś t test.

## Supplemental Information

Document S1. Figures S1–S6

Table S1. SILAC-Based Quantitative Proteomics Analyses, Related to Figure 3 SILAC-based quantitative proteomic analyses shows nearly 2,000 neuronal proteins that do not show significant changes in turnover after ATG5 depletion (first tab) and a selection of proteins that exhibited a reduced degradation rate (second tab).

Table S2. Oligonucleotides Used in This Study, Related to STAR Methods

Document S2. Article plus Supplemental Information

## References

[bib1] Andres-Alonso M., Ammar M.R., Butnaru I., Gomes G.M., Acuña Sanhueza G., Raman R., Yuanxiang P., Borgmeyer M., Lopez-Rojas J., Raza S.A. (2019). SIPA1L2 controls trafficking and local signaling of TrkB-containing amphisomes at presynaptic terminals. Nat. Commun..

[bib2] Ariosa A.R., Klionsky D.J. (2016). Autophagy core machinery: overcoming spatial barriers in neurons. J. Mol. Med. (Berl.).

[bib3] Ashrafi G., Schlehe J.S., LaVoie M.J., Schwarz T.L. (2014). Mitophagy of damaged mitochondria occurs locally in distal neuronal axons and requires PINK1 and Parkin. J. Cell Biol..

[bib4] Azarnia Tehran D., Kuijpers M., Haucke V. (2018). Presynaptic endocytic factors in autophagy and neurodegeneration. Curr. Opin. Neurobiol..

[bib5] Bago R., Malik N., Munson M.J., Prescott A.R., Davies P., Sommer E., Shpiro N., Ward R., Cross D., Ganley I.G., Alessi D.R. (2014). Characterization of VPS34-IN1, a selective inhibitor of Vps34, reveals that the phosphatidylinositol 3-phosphate-binding SGK3 protein kinase is a downstream target of class III phosphoinositide 3-kinase. Biochem. J..

[bib89] Belevich I., Joensuu M., Kumar D., Vihinen H., Jokitalo E. (2016). Microscopy image browser: a platform for segmentation and analysis of multidimensional datasets. PLoS Biol..

[bib6] Berner N., Reutter K.R., Wolf D.H. (2018). Protein Quality Control of the Endoplasmic Reticulum and Ubiquitin-Proteasome-Triggered Degradation of Aberrant Proteins: Yeast Pioneers the Path. Annu. Rev. Biochem..

[bib7] Bezprozvanny I., Kavalali E.T. (2020). Presynaptic endoplasmic reticulum and neurotransmission. Cell Calcium.

[bib8] Bischofberger J., Engel D., Li L., Geiger J.R., Jonas P. (2006). Patch-clamp recording from mossy fiber terminals in hippocampal slices. Nat. Protoc..

[bib9] Branco T., Staras K. (2009). The probability of neurotransmitter release: variability and feedback control at single synapses. Nat. Rev. Neurosci..

[bib10] Cajigas I.J., Will T., Schuman E.M. (2010). Protein homeostasis and synaptic plasticity. EMBO J..

[bib86] Chen T.-W., Wardill T.J., Sun Y., Pulver S.R., Renninger S.L., Baohan A., Schreiter E.R., Kerr R.A., Orger M.B., Jayaraman V. (2013). Ultrasensitive fluorescent proteins for imaging neuronal activity. Nature.

[bib11] Corrochano S., Renna M., Tomas-Zapico C., Brown S.D., Lucas J.J., Rubinsztein D.C., Acevedo-Arozena A. (2012). α-Synuclein levels affect autophagosome numbers in vivo and modulate Huntington disease pathology. Autophagy.

[bib12] de Juan-Sanz J., Holt G.T., Schreiter E.R., de Juan F., Kim D.S., Ryan T.A. (2017). Axonal Endoplasmic Reticulum Ca(2+) Content Controls Release Probability in CNS Nerve Terminals. Neuron.

[bib13] Del Prete D., Checler F., Chami M. (2014). Ryanodine receptors: physiological function and deregulation in Alzheimer disease. Mol. Neurodegener..

[bib87] Eden E., Navon R., Steinfeld I., Lipson D., Yakhini Z. (2009). GOrilla: a tool for discovery and visualization of enriched GO terms in ranked gene lists. BMC Bioinformatics.

[bib14] Friedman L.G., Lachenmayer M.L., Wang J., He L., Poulose S.M., Komatsu M., Holstein G.R., Yue Z. (2012). Disrupted autophagy leads to dopaminergic axon and dendrite degeneration and promotes presynaptic accumulation of α-synuclein and LRRK2 in the brain. J. Neurosci..

[bib15] Galante M., Marty A. (2003). Presynaptic ryanodine-sensitive calcium stores contribute to evoked neurotransmitter release at the basket cell-Purkinje cell synapse. J. Neurosci..

[bib16] Grumati P., Dikic I., Stolz A. (2018). ER-phagy at a glance. J. Cell Sci..

[bib17] Guedes-Dias P., Holzbaur E.L.F. (2019). Axonal transport: Driving synaptic function. Science.

[bib18] Hakim V., Cohen L.D., Zuchman R., Ziv T., Ziv N.E. (2016). The effects of proteasomal inhibition on synaptic proteostasis. EMBO J..

[bib19] Hara T., Nakamura K., Matsui M., Yamamoto A., Nakahara Y., Suzuki-Migishima R., Yokoyama M., Mishima K., Saito I., Okano H., Mizushima N. (2006). Suppression of basal autophagy in neural cells causes neurodegenerative disease in mice. Nature.

[bib20] Haucke V., Neher E., Sigrist S.J. (2011). Protein scaffolds in the coupling of synaptic exocytosis and endocytosis. Nat. Rev. Neurosci..

[bib21] Hayashi S., McMahon A.P. (2002). Efficient recombination in diverse tissues by a tamoxifen-inducible form of Cre: a tool for temporally regulated gene activation/inactivation in the mouse. Dev. Biol..

[bib22] Hernández D.E., Salvadores N.A., Moya-Alvarado G., Catalán R.J., Bronfman F.C., Court F.A. (2018). Axonal degeneration induced by glutamate excitotoxicity is mediated by necroptosis. J. Cell Sci..

[bib23] Hill S.E., Colón-Ramos D.A. (2020). The Journey of the Synaptic Autophagosome: A Cell Biological Perspective. Neuron.

[bib24] Hoffmann S., Orlando M., Andrzejak E., Bruns C., Trimbuch T., Rosenmund C., Garner C.C., Ackermann F. (2019). Light-Activated ROS Production Induces Synaptic Autophagy. J. Neurosci..

[bib25] Honda S., Arakawa S., Nishida Y., Yamaguchi H., Ishii E., Shimizu S. (2014). Ulk1-mediated Atg5-independent macroautophagy mediates elimination of mitochondria from embryonic reticulocytes. Nat. Commun..

[bib26] Irie T., Trussell L.O. (2017). Double-Nanodomain Coupling of Calcium Channels, Ryanodine Receptors, and BK Channels Controls the Generation of Burst Firing. Neuron.

[bib27] Iwasato T., Datwani A., Wolf A.M., Nishiyama H., Taguchi Y., Tonegawa S., Knöpfel T., Erzurumlu R.S., Itohara S. (2000). Cortex-restricted disruption of NMDAR1 impairs neuronal patterns in the barrel cortex. Nature.

[bib28] Jahn R., Fasshauer D. (2012). Molecular machines governing exocytosis of synaptic vesicles. Nature.

[bib29] Kaeser P.S., Regehr W.G. (2017). The readily releasable pool of synaptic vesicles. Curr. Opin. Neurobiol..

[bib30] Kavalali E.T., Jorgensen E.M. (2014). Visualizing presynaptic function. Nat. Neurosci..

[bib31] Ketel K., Krauss M., Nicot A.S., Puchkov D., Wieffer M., Müller R., Subramanian D., Schultz C., Laporte J., Haucke V. (2016). A phosphoinositide conversion mechanism for exit from endosomes. Nature.

[bib32] Khaminets A., Heinrich T., Mari M., Grumati P., Huebner A.K., Akutsu M., Liebmann L., Stolz A., Nietzsche S., Koch N. (2015). Regulation of endoplasmic reticulum turnover by selective autophagy. Nature.

[bib85] Kimura S., Noda T., Yoshimori T. (2007). Dissection of the autophagosome maturation process by a novel reporter protein, tandem fluorescent-tagged LC3. Autophagy.

[bib33] Komatsu M., Waguri S., Chiba T., Murata S., Iwata J., Tanida I., Ueno T., Koike M., Uchiyama Y., Kominami E., Tanaka K. (2006). Loss of autophagy in the central nervous system causes neurodegeneration in mice. Nature.

[bib34] Komatsu M., Wang Q.J., Holstein G.R., Friedrich V.L., Iwata J., Kominami E., Chait B.T., Tanaka K., Yue Z. (2007). Essential role for autophagy protein Atg7 in the maintenance of axonal homeostasis and the prevention of axonal degeneration. Proc. Natl. Acad. Sci. USA.

[bib35] Kononenko N.L., Claßen G.A., Kuijpers M., Puchkov D., Maritzen T., Tempes A., Malik A.R., Skalecka A., Bera S., Jaworski J., Haucke V. (2017). Retrograde transport of TrkB-containing autophagosomes via the adaptor AP-2 mediates neuronal complexity and prevents neurodegeneration. Nat. Commun..

[bib88] Kremer J.R., Mastronarde D.N., McIntosh J.R. (1996). Computer visualization of three-dimensional image data using IMOD. J Struct BIol..

[bib84] Lee I.H., Cao L., Mostoslavsky R., Lombard D.B., Liu J., Bruns N.E., Tsokos M., Alt F.W., Finkel T. (2008). A role for the NAD-dependent deacetylase Sirt1 in the regulation of autophagy. Proc Natl Acad Sci USA.

[bib36] Liang J.R., Lingeman E., Ahmed S., Corn J.E. (2018). Atlastins remodel the endoplasmic reticulum for selective autophagy. J. Cell Biol..

[bib37] Lieberman O.J., Frier M.D., McGuirt A.F., Griffey C.J., Rafikian E., Yang M., Yamamoto A., Borgkvist A., Santini E., Sulzer D. (2020). Cell-type-specific regulation of neuronal intrinsic excitability by macroautophagy. eLife.

[bib38] Lindhout F.W., Cao Y., Kevenaar J.T., Bodzęta A., Stucchi R., Boumpoutsari M.M., Katrukha E.A., Altelaar M., MacGillavry H.D., Hoogenraad C.C. (2019). VAP-SCRN1 interaction regulates dynamic endoplasmic reticulum remodeling and presynaptic function. EMBO J..

[bib39] Maday S., Holzbaur E.L. (2014). Autophagosome biogenesis in primary neurons follows an ordered and spatially regulated pathway. Dev. Cell.

[bib40] Maday S., Holzbaur E.L. (2016). Compartment-Specific Regulation of Autophagy in Primary Neurons. J. Neurosci..

[bib41] Maday S., Wallace K.E., Holzbaur E.L. (2012). Autophagosomes initiate distally and mature during transport toward the cell soma in primary neurons. J. Cell Biol..

[bib42] Mata G., Heras J., Morales M., Romero A., Rubio J., Fred A., Gamboa H. (2016). SynapCountJ: A tool for analyzing synaptic densities in neurons. Proceedings of the 9th International Joint Conference on Biomedical Enginerring Systems and Technologies.

[bib43] Moreau K., Fleming A., Imarisio S., Lopez Ramirez A., Mercer J.L., Jimenez-Sanchez M., Bento C.F., Puri C., Zavodszky E., Siddiqi F. (2014). PICALM modulates autophagy activity and tau accumulation. Nat. Commun..

[bib44] Murdoch J.D., Rostosky C.M., Gowrisankaran S., Arora A.S., Soukup S.F., Vidal R., Capece V., Freytag S., Fischer A., Verstreken P. (2016). Endophilin-A Deficiency Induces the Foxo3a-Fbxo32 Network in the Brain and Causes Dysregulation of Autophagy and the Ubiquitin-Proteasome System. Cell Rep..

[bib45] Murthy V.N., De Camilli P. (2003). Cell biology of the presynaptic terminal. Annu. Rev. Neurosci..

[bib46] Nanou E., Catterall W.A. (2018). Calcium Channels, Synaptic Plasticity, and Neuropsychiatric Disease. Neuron.

[bib47] Neher E., Sakaba T. (2008). Multiple roles of calcium ions in the regulation of neurotransmitter release. Neuron.

[bib48] Nicoll R.A., Schmitz D. (2005). Synaptic plasticity at hippocampal mossy fibre synapses. Nat. Rev. Neurosci..

[bib49] Nikoletopoulou V., Tavernarakis N. (2018). Regulation and Roles of Autophagy at Synapses. Trends Cell Biol..

[bib50] Nishida Y., Arakawa S., Fujitani K., Yamaguchi H., Mizuta T., Kanaseki T., Komatsu M., Otsu K., Tsujimoto Y., Shimizu S. (2009). Discovery of Atg5/Atg7-independent alternative macroautophagy. Nature.

[bib51] Nixon R.A. (2013). The role of autophagy in neurodegenerative disease. Nat. Med..

[bib52] Okerlund N.D., Schneider K., Leal-Ortiz S., Montenegro-Venegas C., Kim S.A., Garner L.C., Waites C.L., Gundelfinger E.D., Reimer R.J., Garner C.C. (2017). Bassoon Controls Presynaptic Autophagy through Atg5. Neuron.

[bib53] Ravikumar B., Vacher C., Berger Z., Davies J.E., Luo S., Oroz L.G., Scaravilli F., Easton D.F., Duden R., O’Kane C.J., Rubinsztein D.C. (2004). Inhibition of mTOR induces autophagy and reduces toxicity of polyglutamine expansions in fly and mouse models of Huntington disease. Nat. Genet..

[bib54] Ravikumar B., Imarisio S., Sarkar S., O’Kane C.J., Rubinsztein D.C. (2008). Rab5 modulates aggregation and toxicity of mutant huntingtin through macroautophagy in cell and fly models of Huntington disease. J. Cell Sci..

[bib55] Ravikumar B., Sarkar S., Davies J.E., Futter M., Garcia-Arencibia M., Green-Thompson Z.W., Jimenez-Sanchez M., Korolchuk V.I., Lichtenberg M., Luo S. (2010). Regulation of mammalian autophagy in physiology and pathophysiology. Physiol. Rev..

[bib56] Rizzoli S.O. (2014). Synaptic vesicle recycling: steps and principles. EMBO J..

[bib57] Saheki Y., De Camilli P. (2017). Endoplasmic Reticulum-Plasma Membrane Contact Sites. Annu. Rev. Biochem..

[bib58] Sarkar S., Perlstein E.O., Imarisio S., Pineau S., Cordenier A., Maglathlin R.L., Webster J.A., Lewis T.A., O’Kane C.J., Schreiber S.L., Rubinsztein D.C. (2007). Small molecules enhance autophagy and reduce toxicity in Huntington’s disease models. Nat. Chem. Biol..

[bib59] Sato I., Kamiya H. (2011). Assessing the roles of presynaptic ryanodine receptors and adenosine receptors in caffeine-induced enhancement of hippocampal mossy fiber transmission. Neurosci. Res..

[bib60] Schneggenburger R., Meyer A.C., Neher E. (1999). Released fraction and total size of a pool of immediately available transmitter quanta at a calyx synapse. Neuron.

[bib61] Schuck S., Prinz W.A., Thorn K.S., Voss C., Walter P. (2009). Membrane expansion alleviates endoplasmic reticulum stress independently of the unfolded protein response. J. Cell Biol..

[bib62] Schulz P.E. (1997). Long-term potentiation involves increases in the probability of neurotransmitter release. Proc. Natl. Acad. Sci. USA.

[bib63] Scullin C.S., Partridge L.D. (2010). Contributions of SERCA pump and ryanodine-sensitive stores to presynaptic residual Ca2+. Cell Calcium.

[bib64] Shehata M., Matsumura H., Okubo-Suzuki R., Ohkawa N., Inokuchi K. (2012). Neuronal stimulation induces autophagy in hippocampal neurons that is involved in AMPA receptor degradation after chemical long-term depression. J. Neurosci..

[bib65] Shen H., Zhu H., Panja D., Gu Q., Li Z. (2020). Autophagy controls the induction and developmental decline of NMDAR-LTD through endocytic recycling. Nat. Commun..

[bib66] Shimizu H., Fukaya M., Yamasaki M., Watanabe M., Manabe T., Kamiya H. (2008). Use-dependent amplification of presynaptic Ca2+ signaling by axonal ryanodine receptors at the hippocampal mossy fiber synapse. Proc. Natl. Acad. Sci. USA.

[bib67] Sola E., Prestori F., Rossi P., Taglietti V., D’Angelo E. (2004). Increased neurotransmitter release during long-term potentiation at mossy fibre-granule cell synapses in rat cerebellum. J. Physiol..

[bib68] Soukup S.F., Verstreken P. (2017). EndoA/Endophilin-A creates docking stations for autophagic proteins at synapses. Autophagy.

[bib69] Soukup S.F., Kuenen S., Vanhauwaert R., Manetsberger J., Hernández-Díaz S., Swerts J., Schoovaerts N., Vilain S., Gounko N.V., Vints K. (2016). A LRRK2-Dependent EndophilinA Phosphoswitch Is Critical for Macroautophagy at Presynaptic Terminals. Neuron.

[bib70] Stavoe A.K.H., Holzbaur E.L.F. (2019). Autophagy in Neurons. Annu. Rev. Cell Dev. Biol..

[bib71] Südhof T.C. (2013). Neurotransmitter release: the last millisecond in the life of a synaptic vesicle. Neuron.

[bib72] Unni V.K., Zakharenko S.S., Zablow L., DeCostanzo A.J., Siegelbaum S.A. (2004). Calcium release from presynaptic ryanodine-sensitive stores is required for long-term depression at hippocampal CA3-CA3 pyramidal neuron synapses. J. Neurosci..

[bib73] Vijayan V., Verstreken P. (2017). Autophagy in the presynaptic compartment in health and disease. J. Cell Biol..

[bib74] Waites C.L., Leal-Ortiz S.A., Okerlund N., Dalke H., Fejtova A., Altrock W.D., Gundelfinger E.D., Garner C.C. (2013). Bassoon and Piccolo maintain synapse integrity by regulating protein ubiquitination and degradation. EMBO J..

[bib75] Wan H., Wang Q., Chen X., Zeng Q., Shao Y., Fang H., Liao X., Li H.S., Liu M.G., Xu T.L. (2020). WDR45 contributes to neurodegeneration through regulation of ER homeostasis and neuronal death. Autophagy.

[bib76] Wang Y., Qin Z.H. (2010). Molecular and cellular mechanisms of excitotoxic neuronal death. Apoptosis.

[bib77] Wang T., Martin S., Papadopulos A., Harper C.B., Mavlyutov T.A., Niranjan D., Glass N.R., Cooper-White J.J., Sibarita J.B., Choquet D. (2015). Control of autophagosome axonal retrograde flux by presynaptic activity unveiled using botulinum neurotoxin type a. J. Neurosci..

[bib78] Weisskopf M.G., Nicoll R.A. (1995). Presynaptic changes during mossy fibre LTP revealed by NMDA receptor-mediated synaptic responses. Nature.

[bib79] Westrate L.M., Lee J.E., Prinz W.A., Voeltz G.K. (2015). Form follows function: the importance of endoplasmic reticulum shape. Annu. Rev. Biochem..

[bib80] Williams A., Jahreiss L., Sarkar S., Saiki S., Menzies F.M., Ravikumar B., Rubinsztein D.C. (2006). Aggregate-prone proteins are cleared from the cytosol by autophagy: therapeutic implications. Curr. Top. Dev. Biol..

[bib81] Yang Y., Calakos N. (2013). Presynaptic long-term plasticity. Front. Synaptic Neurosci..

[bib82] Zhang H., Hu J. (2016). Shaping the Endoplasmic Reticulum into a Social Network. Trends Cell Biol..

[bib83] Zucker R.S., Regehr W.G. (2002). Short-term synaptic plasticity. Annu. Rev. Physiol..

