# Neuronal Autophagy Regulates Presynaptic Neurotransmission by Controlling the Axonal Endoplasmic Reticulum

**DOI:** 10.1016/j.neuron.2022.01.029

**Published:** 2022-02-16

**Authors:** Marijn Kuijpers, Gaga Kochlamazashvili, Alexander Stumpf, Dmytro Puchkov, Aarti Swaminathan, Max Thomas Lucht, Eberhard Krause, Tanja Maritzen, Dietmar Schmitz, Volker Haucke

(Neuron *109*, 299–313.e1–e9; January 20, 2021)

The authors noticed two inadvertent errors in their publication. In Figure 6G, the DAPI channels of the wild-type and knockout brain slices were erroneously swapped. In Figure S5A, an incorrect representative immunoblot for calnexin was shown that originated from a different experiment. The corrected Figures 6G and S5A are shown below. These corrections do not affect the analysis of data or statements in the text and do not affect the conclusion of this paper. The authors sincerely apologize for both errors.

**Figure 6G:**
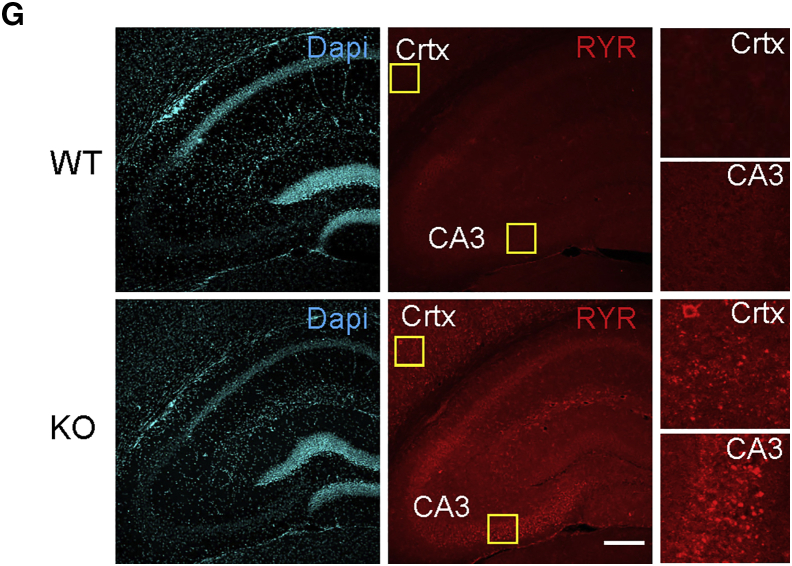
Increased RyR-Mediated Calcium Release Underlies Elevated Neurotransmission in ATG5 KO Neurons

**Figure S5A:**
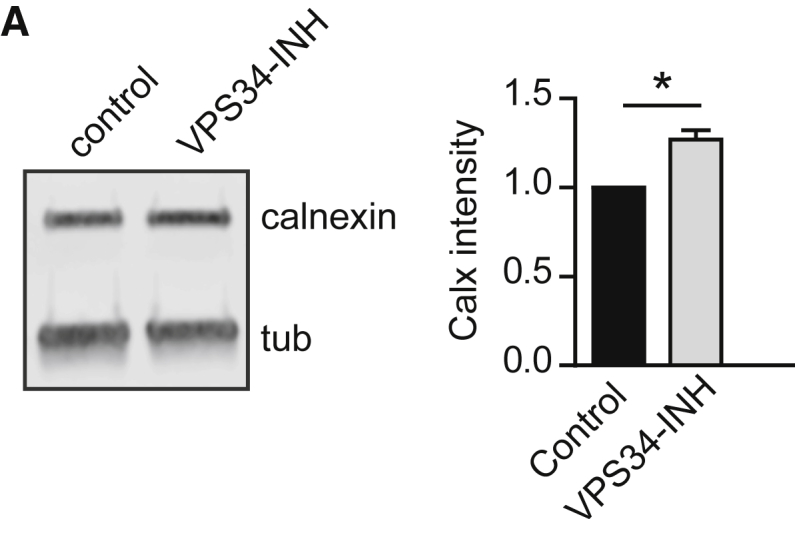
Loss of ATG5 does not affect mitochondrial acidification, axonal lipid levels or ER integrity

