# Supplementary material for: Neuronal Autophagy Regulates Presynaptic Neurotransmission by Controlling the Axonal Endoplasmic Reticulum
Source: Neuron. 2021 Jan 20;109(2):299–313.e9. doi: 10.1016/j.neuron.2020.10.005 (PMC7837115; doi:10.1016/j.neuron.2020.10.005)
Supplement: Document S1. Figures S1–S6 [file mmc1.pdf]

Neuron, Volume *109*

Supplemental Information

Neuronal Autophagy Regulates Presynaptic Neurotransmission by Controlling the Axonal Endoplasmic Reticulum

Marijn Kuijpers, Gaga Kochlamazashvili, Alexander Stumpf, Dmytro Puchkov, Aarti Swaminathan, Max Thomas Lucht, Eberhard Krause, Tanja Maritzen, Dietmar Schmitz, and Volker Haucke

Supplementary File for

Neuronal autophagy regulates presynaptic neurotransmission by controlling the axonal endoplasmic reticulum

Marijn Kuijpers, Gaga Kochlamazashvili, Alexander Stumpf, Dmytro Puchkov, Aarti Swaminathan, Max Thomas Lucht, Eberhard Krause, Tanja Maritzen, Dietmar Schmitz and Volker Haucke

Inventory of supplementary items

Supplementary Figures

Supplementary figure S1 (related to Figure 1) | Characterization of ATG5-iKO brains

Supplementary figure S2 (related to Figure 1) | Altered synaptic properties at Schaffer collateral and mossy fiber synapses in ATG5-cKO mice

Supplementary figure S3 (related to Figures 2 and 3) | Accumulation of proteins in ATG5- iKO neurons

Supplementary figure S4 (related to Figure 4) | Loss of autophagy leads to axonal ER accumulation but does not affect Golgi, lysosomes or mitochondria

Supplementary figure S5 (related to Figures 5 and 6) | Loss of ATG5 does not affect mitochondrial acidification, axonal lipid levels or ER integrity

Supplementary figure S6 (related to Figure 6) | ATG5 KO brains have increased RYR levels and inhibiting RYR function rescues elevated neurotransmission in ATG5-iKO neurons

Supplementary figures

S1 (Related to Figure 1)

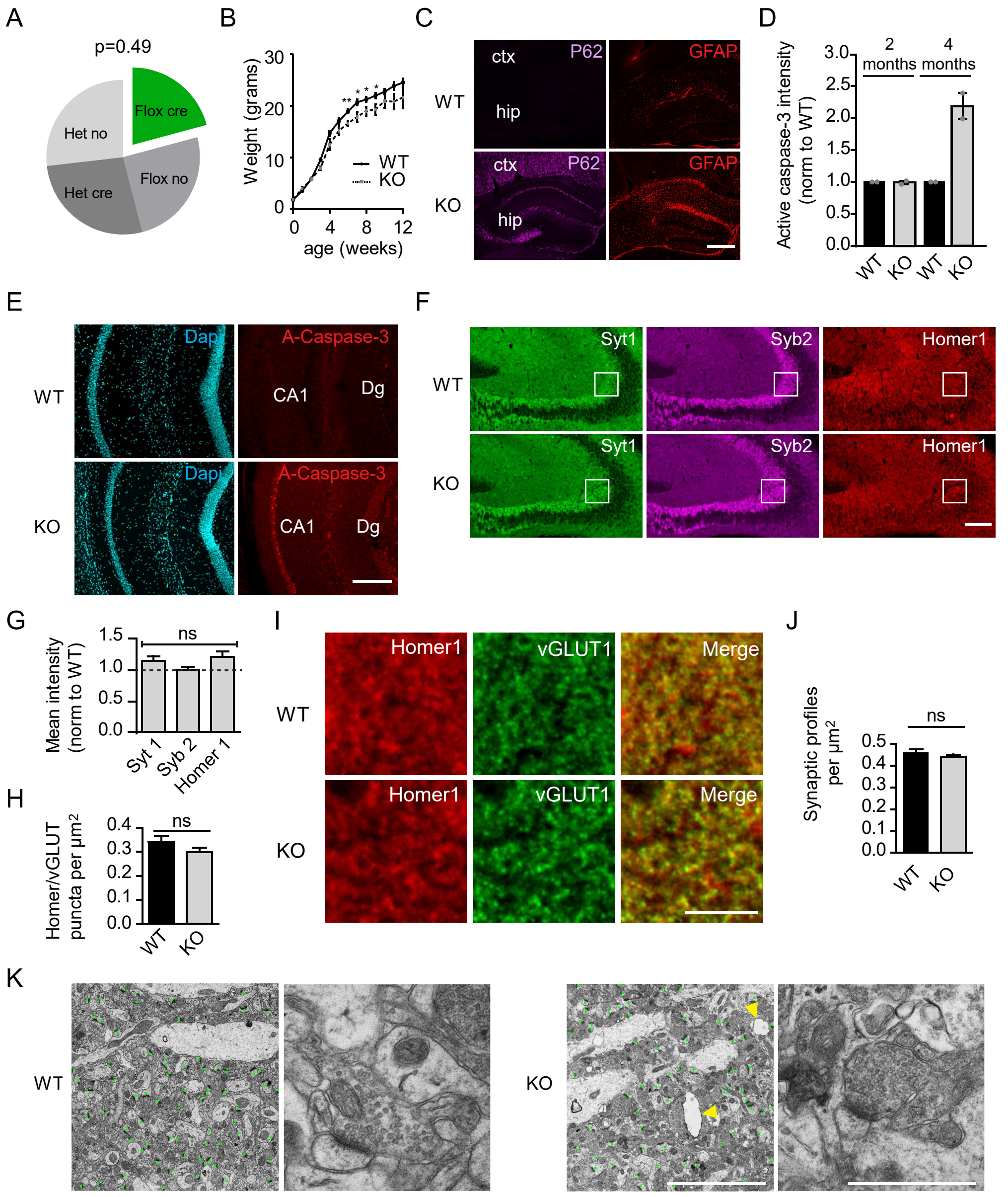

Supplementary figure S1 (related to Figure 1) | Characterization of ATG5-cKO brains

(A) Birth ratios of ATG5$^{flox/flox}$ x ATG5$^{flox/-}$;EMX1-*Cre* matings with chi-squared analysis. n=235 mice.

(B) Growth curves of ATG5-cKO (n=4-44) mice and their littermate controls (n=16-103); t-test for individual timepoints.

(C) GFAP and p62 immunostaining in 6-7 week-old control and ATG5-cKO brain slices. Ctx=cortex, hip=hippocampus. Scale bar, 400 μm.

(D,E) Active caspase-3 immunostaining in young (<2months) versus "old" (4 months) control and ATG5-cKO brain slices. (D) Quantification of active caspase-3 intensity in the CA1 area. Slices taken from 2 mice. The mean value for the controls are set to 1, and the mean value for the KO is expressed relative to this. Values of single mice are plotted as individual points in the graph. (E) An example of active caspase-3 immunostaining in "old" (4 months) mice. Dg=dentate gyrus. Scale bar, 200 μm.

(F,G) Immunostaining in 6-7 week-old control and ATG5-cKO brain slices. (F) Images of hippocampal sections depicting typical levels of synaptotagmin 1 (Syt1), synaptobrevin 2 (Syb2) and Homer 1 immunoreactivities. White boxes indicate area taken for quantification (G) Expression levels of Syt1, Syb2 and Homer 1 are not significantly different in ATG5-cKO compared to control littermates. The mean value for the controls was set to 1, and the mean value for the KO is expressed relative to this. Slices taken from 3 mice; one-sample t-test. Scale bar, 100 μm.

(H,I) Immunostaining of pre-and postsynaptic markers in CA1 area of 2 months old control and ATG5-cKO brain slices. (H) Number of Homer 1/ vGLUT1-positive puncta are not significantly different in ATG5-cKO compared to control littermates. Slices taken from 4 mice, 6 20x20 μm

ROIs are analyzed per mouse; t-test. (I) Example images of hippocampal sections (CA1) depicting pre-synaptic (vGLUT1) and post-synaptic (Homer 1) colocalization. Scale bar, 10 μm,

(J) Number of synapses in mouse CA1 area analyzed by electron microscopy. n=10 (WT) or 12 (KO) micrographs representing ~900 synapses per genotype, 1 experiment; t-test.

(K) Representative electron micrographs of synaptic profile counts. Green colored areas indicate synaptic profiles. Yellow arrows point to holes indicative of minor neuron loss in ATG5-cKO CA1 area. Scale bar, 100 μm (left) or 1 μm (right). ns: not significant, $*p < 0.05$; $**p < 0.01$.

S2 (Related to Figure 1)

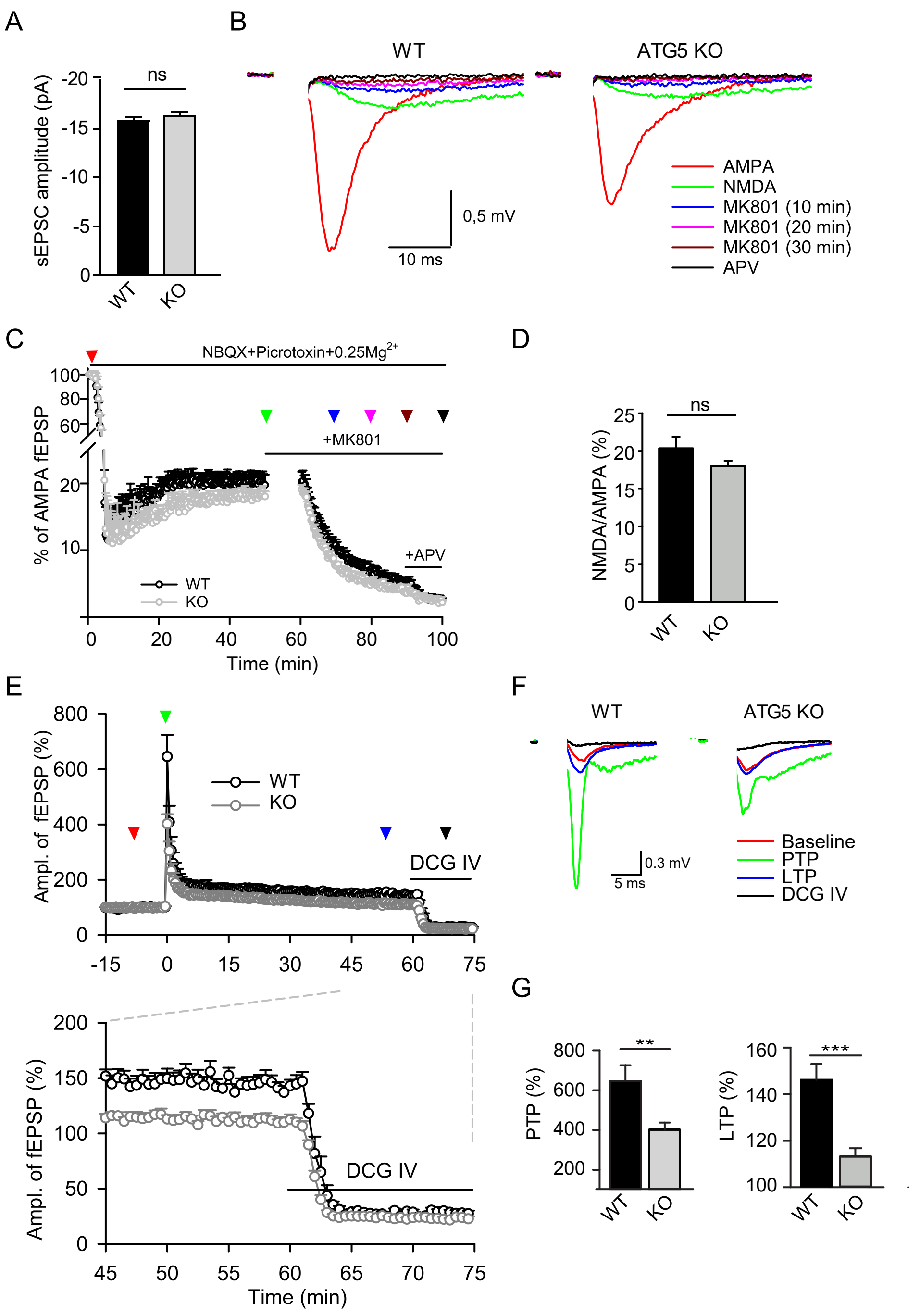

Supplementary figure S2 (related to Figure 1) | Altered synaptic properties at Schaffer collateral and mossy fiber synapses in ATG5-cKO mice

(A) Summary result of averaged sEPSC amplitude. n=17 (WT) or 21 (KO) slices from 7 (WT) or 6 (KO) animals; Mann–Whitney test. (B) Representative WT and KO fEPSPs show AMPA and NMDA receptor mediated responses before and after application of antagonists. Traces are color-coded according to arrowheads in panel C. (C) NMDA receptor-mediated fEPSPs were isolated using the AMPA/kainite receptor antagonist NBQX (10µM) and the $GABA_A$/glycine receptor antagonist picrotoxin (50µM). Initial AMPA receptor-mediated responses were taken as 100%. The non-competitive irreversible NMDA receptor antagonist MK801 (30µM) was applied for 10 min before and 30 min during stimulation to measure release probability. At the end of every experiment the potent NMDA receptor antagonist APV (50µM) was applied. n=12 (WT) or 10 (KO) slices from 7 (WT) and 6 (KO) animals, respectively; a break in the y-axis is introduced between 25 and 50 to better visualize the curves. (D) The ratio of NMDA/AMPA responses in ATG5-cKO *vs.* WT mice is unaltered. Data taken from figure C. t-test (P=0.181, n.s.). (E) Decreased post-tetanic and long-term potentiation induced by 5xHFS at mossy fiber-CA3 synapses in Atg5-cKO (n=8 slices, 6 mice) as compared to control mice (n=6 slices, 5 mice). Only responses inhibited by 70-80% and more were assumed to be elicited by mossy fiber synapses. The mean amplitudes of fEPSPs recorded between -15 to 0 min was taken as 100%. (F) Representative MF-fEPSP traces (F) are collected before (red), immediately after LTP induction (green), 45-60 min after LTP induction (blue) and after application of the agonist of type II metabotropic glutamate receptors DCG IV (2 µM; black). Traces are color-coded according to arrowheads in panel E. (G) PTP and LTP levels measured immediately after induction (WT; 645.6 ± 79.4 and Atg5-cKO;

402.4 ± 34.9) and between 45–60 min (WT; 146.2 ± 6.7 and Atg5-cKO; 113.2 ± 3.6), respectively, show reduced facilitation in Atg5-cKO mice; unpaired t-test.

All data represent mean ± SEM, ns: not significant, $**p < 0.01$; $***p < 0.001$.

S3 (Related to Figure 2 and 3)

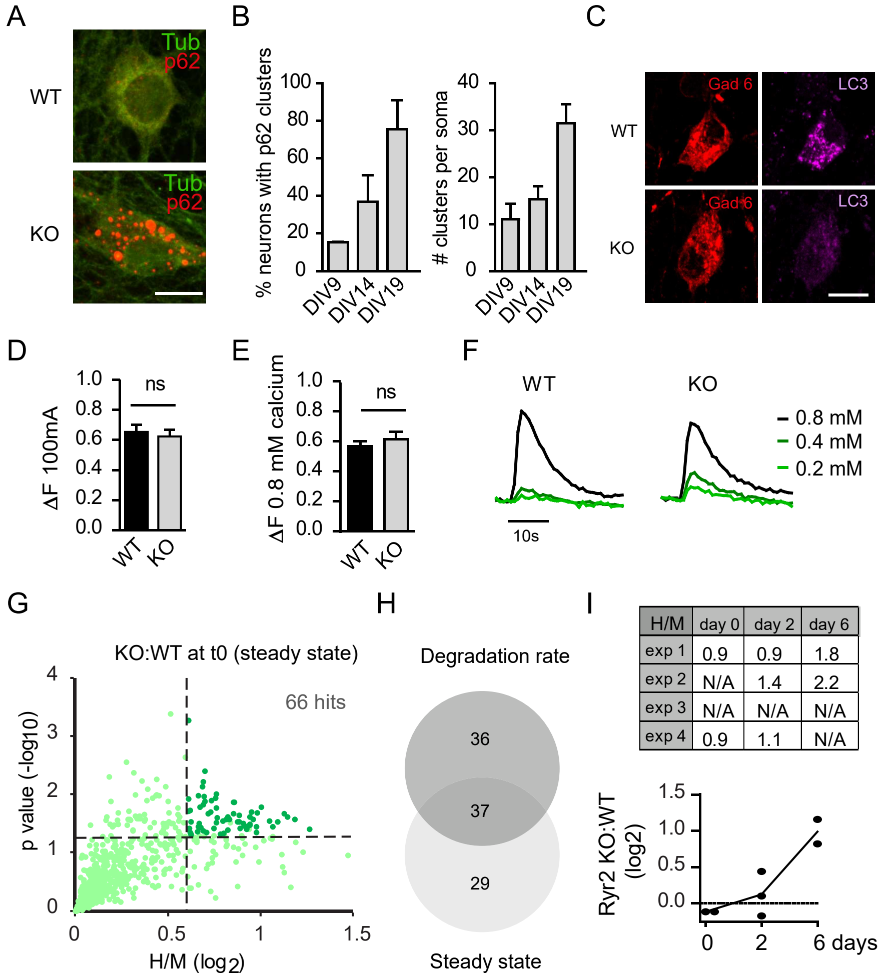

H/M	day 0	day 2	day 6
exp 1	0.9	0.9	1.8
exp 2	N/A	1.4	2.2
exp 3	N/A	N/A	N/A
exp 4	0.9	1.1	N/A

Supplementary figure S3 (related to Figures 2 and 3) | Accumulation of proteins in ATG5-iKO neurons

(A) Representative confocal images of WT and ATG5-iKO hippocampal neurons, immunostained for β3-tubulin and p62. Scale bar, 10 μm. (B) Somatic p62 accumulation in KO neurons increases over time, indicated by % neurons that show p62 clusters (n=2 independent experiments, 200 cells per timepoint) and number of clusters per neuron (n=20 cells). (C) ATG5-iKO inhibitory hippocampal neurons show deficient LC3-positive puncta formation upon bafilomycin treatment (10nm, 4 hours). Scale bar, 10 μm. (D) Maximal fluorescent peak of synaptophysin–pHluorin–expressing neurons at 100 mA (Fmax, 60AP). n=28-30 cells, 4 independent experiments; unpaired t-test. See also Figure 2H.

(E) Maximal fluorescent peak of Synaptophysin–pHluorin–expressing neurons at 0.8 mM calcium (F_{max}, 60AP). n=28-30 cells, 4 independent experiments; unpaired t-test. Data represent mean ± SEM, ns: not significant. n=21 cells, 20 boutons per cell, 3 independent experiments; t-test.. See also Figure 2I.

(F) Example traces (average from representative experiment) showing a calcium-dependent decrease in pHluorin signal in WT and KO hippocampal synapses.

(G) Comparisons of H/M (KO/WT) ratios obtained for proteins of neuron CGN cultures that were lysed at t=0 (DIV14, no replacement with unlabeled medium). Instead of differences in turnover (see Figure 3D for pulsed SILAC) this graph rather shows the accumulation of proteins in the ATG5- iKO neurons (defined as KO/WT>1.5 and p<0.05, dotted lines).

(H) Overlap of protein hits found in steady state SILAC vs pulsed SILAC

(I) Ryanodine Receptor 2 (RyR2) was not detected in all SILAC conditions (and therefore not one of 1753 detected proteins) but plotting of H/M (WT/KO) ratios of single conditions (black dots, see table for H/M ratio values) shows a clear trend of slower RyR2 degradation in ATG5-iKO neurons. Data represent mean ± SEM, ns: not significant.

S4 (Related to Figure 4)

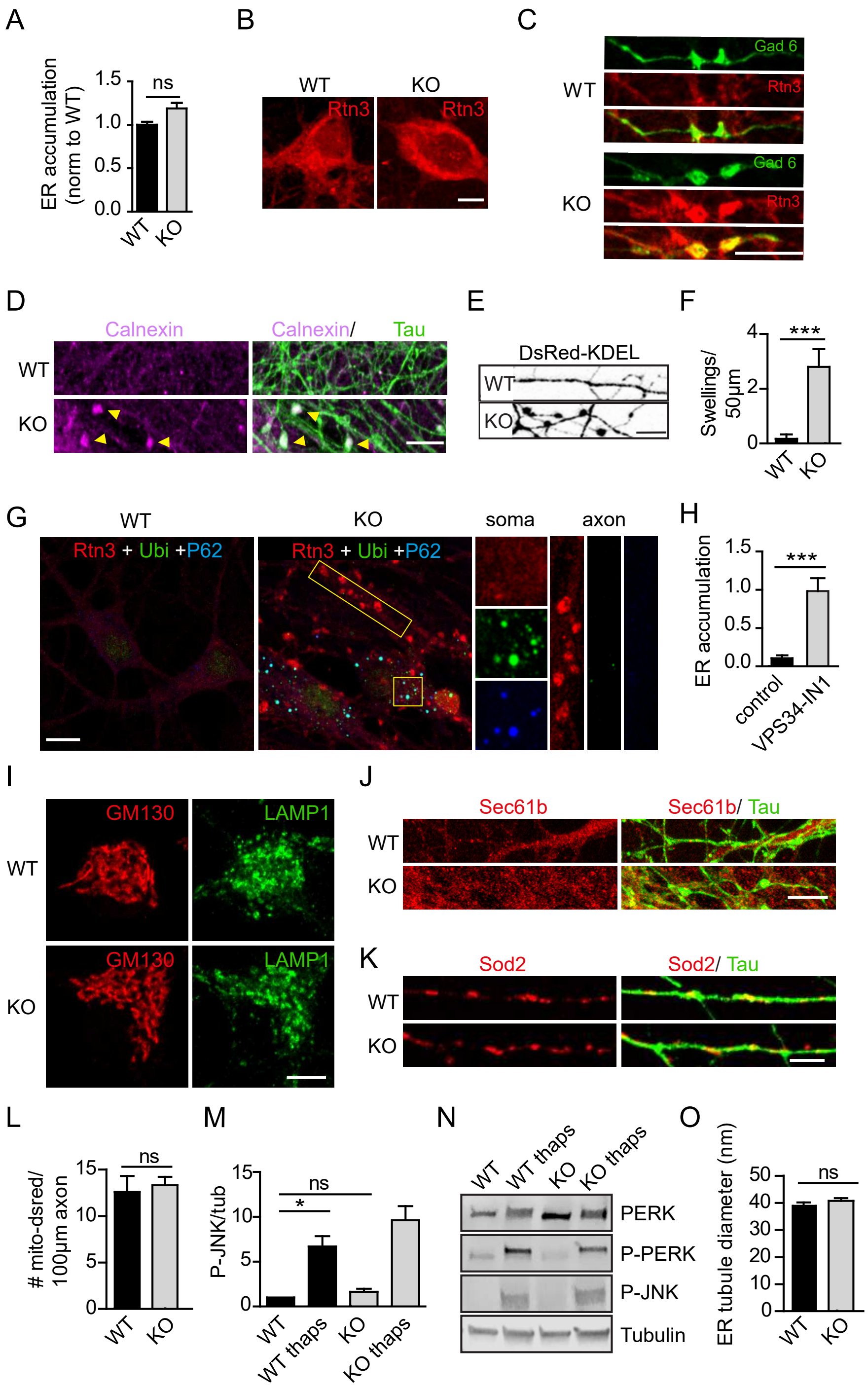

Supplementary figure S4 (related to Figure 4) | Loss of autophagy leads to axonal ER accumulation but does not affect Golgi, lysosomes or mitochondria

(A) Quantification of Rtn3 intensity levels in the soma of WT and ATG5-iKO hippocampal neurons. n=67 somata per genotype from two independent experiments; unpaired t-test.

(B) Representative confocal images of somas of WT and ATG5-iKO hippocampal neuron immunostained for ER marker Rtn3.

(C) Representative confocal images of ER accumulation in ATG5-iKO inhibitory (Gad6 positive) synapses. Scale bar, 5 µm.

(D) Representative confocal images of WT and ATG5-iKO hippocampal neurons immunostained for ER marker Calnexin and axonal marker Tau. Yellow arrows indicate ER accumulations in KO axons.

(E,F) WT and ATG5-iKO hippocampal neurons transfected with DsRed-KDEL. (B) Representative confocal images of DsRed-KDEL positive axons. (C) Quantification of amount of KDEL positive swellings. n=12 (WT) or n=10 (KO) images, 1 experiment; unpaired t-test.

(G) Hippocampal neurons stained for Rtn3, Ubiquitin (Ubi) and p62. Enlarged regions (yellow boxes) show p62 and ubiquitin accumulations in soma and Rtn3 accumulation in axons.

(H) Quantification of Rtn3 in control and VPS34-IN1 (1 µM, 24 hours) treated neurons, expressed as axonal area (Tau positive, not shown) covered with Rtn3 accumulations. n=16-17 images, 1 experiment; unpaired t-test.

(I) Representative confocal images of WT and ATG5-iKO hippocampal neurons immunostained for Golgi marker GM130 and lysosome marker LAMP1.

(J) Representative confocal images of WT and ATG5-iKO hippocampal neurons immunostained for rough ER marker Sec61.

(K-L) No apparent differences between WT and ATG5-iKO neurons in immunostainings and transfection of mitochondrial markers (H) Number of axonal mitochondria quantified in WT and AT5 KO neurons transfected with dsred-mito, n=3 experiments, 26 images per condition; paired t-test

(M) Quantification of JNK phosphorylation (p-JNK) in WT and ATG5-iKO neuron lysates, with and without thapsigargin treatment to induce ER stress (thaps, 1 μM). Conditions are compared to WT, values for WT were set to 1. n=4 experiments; one-sample t-test. See also Figure 4F.

(N) Representative immunoblots of lysates from WT and KO neuron cultures, showing PERK, p-PERK, p-JNK and tubulin content.

(O) ER tubule diameter measured in electron micrographs of WT and ATG5-iKO hippocampal neurons. n=61 tubules from 13 images (WT) or n=62 tubules from 8 images (KO); unpaired t-test.

Scale bars, 5 μm. All data represent mean ± SEM, ns: not significant, $*p < 0.05$; $***p < 0.001$.

S5 (Related to Figure 5 and 6)

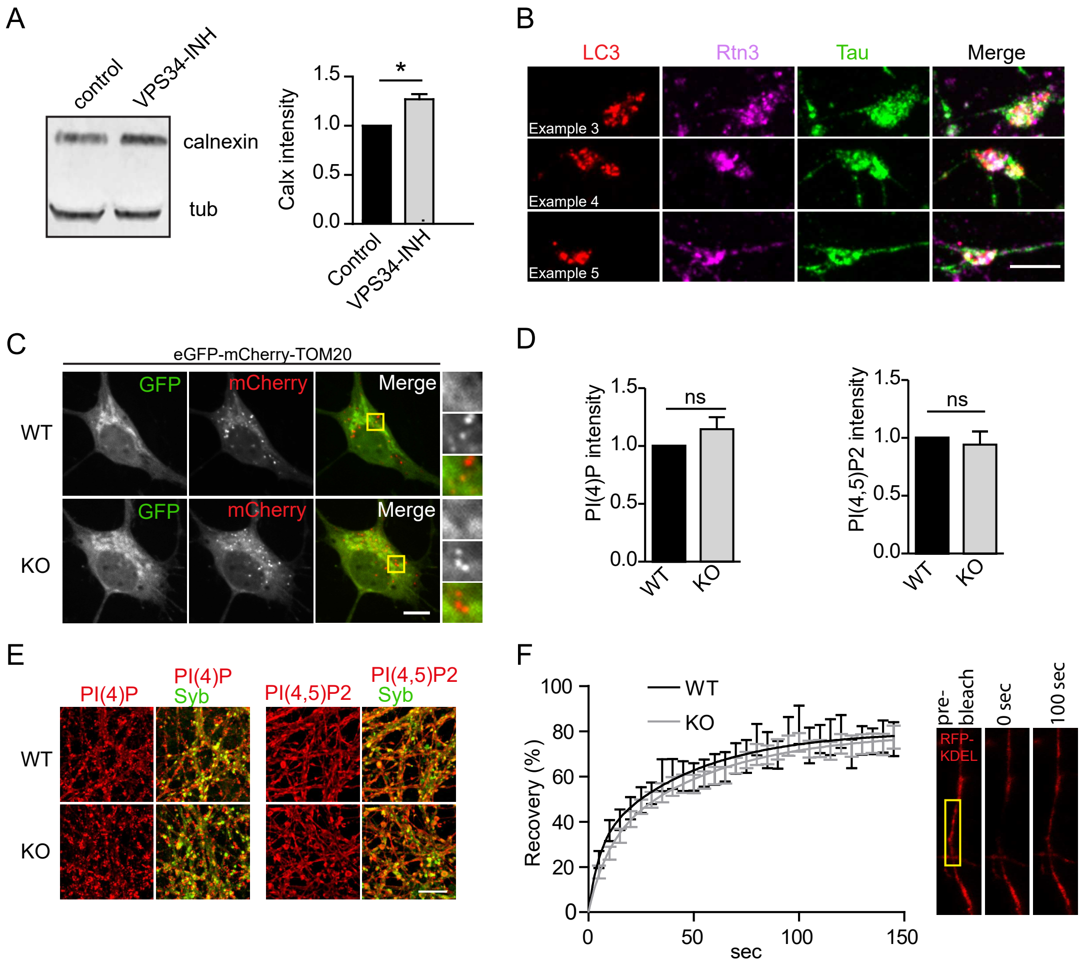

Supplementary figure S5 (related to Figures 5 and 6) | Loss of ATG5 does not affect mitochondrial acidification, axonal lipid levels or ER integrity

(A) Inhibiting autophagy by VPS34 inhibitor treatment (1 μM, 24 hours) leads to Calnexin accumulation. Values for WT were set to 1. n=4 independent experiments; one-sample t-test.

(B) Additional examples of VPS34-IN1 washout experiment described in Figure 5G. Hippocampal axons immunostained for endogenous LC3, Reticulon 3 (Rtn3) and Tau. Scale bar, 5 μm.

(C) Representative confocal images of hippocampal neurons transfected with eGFP-mCherry-TOM20. eGFP is quenched as a result of low pH, causing a switch from GFP+/mCherry+ to GFP−/mCherry+ during lysosomal degradation of mitochondria. Yellow boxes indicate magnifications shown on the right. Scale bar, 5 μm. See Figure 5J for quantification.

(D) Quantification of PI(4)P and PI(4,5)P_2 immunostainings in WT and ATG5-iKO hippocampal synapses (marked by Synaptobrevin 2 immunostaining). The mean values for the WT are set to 1. n=3 (PI(4)P) or n=4 (PI(4,5)P2) independent experiments; one-sample t-test.

(E) Representative confocal images of PI(4)P and PI(4,5)P_2 immunostainings in WT and ATG5-iKO hippocampal cultures. Scale bar, 10 μm.

(F) Fluorescent recovery plots showing the rates of DsRed-KDEL recovery in axons of control neurons and ATG5-iKO neurons. Fluorescent intensity was normalized to intensity before bleaching. n=15 (WT) or 21 (KO) axons, the black and grey curves are the fit of a nonlinear regression model to the experimental data. Images on the right show a time series example, the photobleached area is indicated with a yellow box.

All data represent mean ± SEM, ns: not significant, $*p < 0.05$.

S6 (Related to Figure 6)

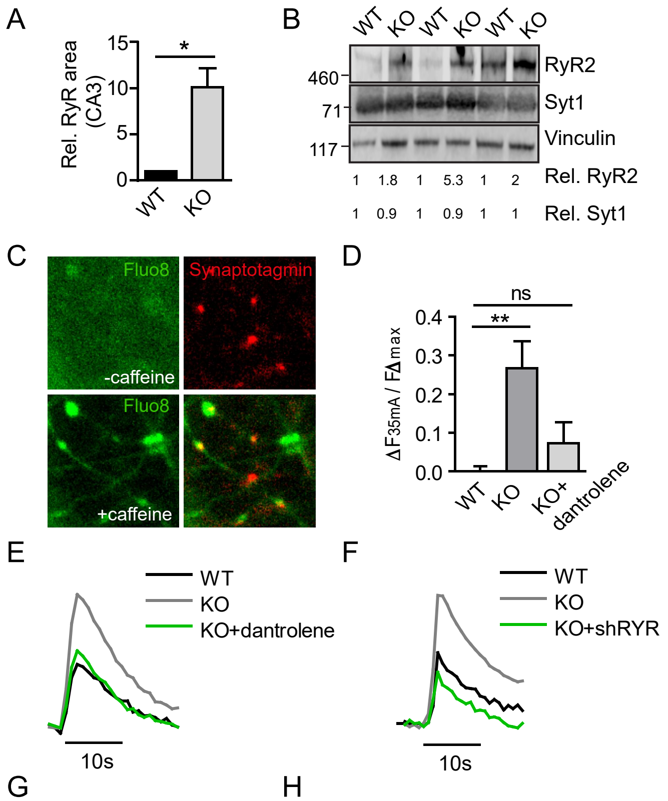

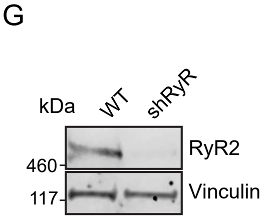

H

shRNA	Axonal ER accumulation
FIP200	yes
Reticulon-3	no
FAM134A	no
FAM134B	no
CCPG1	no
TEX264	no
Atlastin-3	no

Supplementary figure S6 (related to Figure 6) | ATG5 KO brains have increased RYR levels and inhibiting RYR function rescues elevated neurotransmission in ATG5-iKO neurons

(A) Ryanodine Receptor immunoreactivity in hippocampal CA3 area of WT and ATG5-cKO mice. n=3; one-sample t-test. See also Figure 6E.

(B) Immunoblots of WT and ATG5-cKO brain lysates showing increased Ryanodine Receptor 2 (RyR2) levels and no difference in synaptotagmin 1 (Syt1) levels. Density measurements of indicated proteins (normalized to housekeeping protein vinculin) are indicated below the blots.

(C) Example image of a Fluo-8 calcium response showing caffeine-induced calcium release from the ER in ATG5-iKO synapses and axons. Uptake of fluorescently tagged Synaptotagmin 1 antibodies (red) was used to mark synapses and axons in live hippocampal neuron cultures. See also Fig 6H,I,K.

(D) Detection of exocytosis using Synaptophysin-pHluorin in WT and ATG5-iKO hippocampal neurons. Graph showing mean normalized peak fluorescence upon a 35mA stimulation. Dantrolene (10 μM), a Ryanodine receptor inhibitor, rescues increased responses in ATG5-iKO neurons. Values per cell are normalized to the corresponding maximal fluorescent peak at 100 mA (Fmax). n=18-22 cells, 3 experiments; one-way ANOVA with Tukey's post-test.

(E,F) Average traces showing stimulus dependent decreases in Synaptophysin-pHluorin signal in WT and ATG5-iKO synapses. Both dantrolene treatment (E) and lentivirus-mediated knockdown of RYR (F) decrease exocytosis in ATG5-iKO neurons.

(G) Immunoblot of lysates from neurons infected with scrambled or (pan)RYR-shRNA virus, probed for RYR2 and vinculin antibodies.

(H) Lentiviral knockdown (KD) screen for the indicated ER-phagy adaptors. None of the adaptor KD conditions (each consisting of 2-4 different shRNA sequences) lead to axonal ER increases measured by Calnexin or Rtn3 immunostainings.

All data represent mean ± SEM, ns: not significant, $*p < 0.05$; $**p < 0.01$.
