# Supplementary material for: Neuronal Autophagy Regulates Presynaptic Neurotransmission by Controlling the Axonal Endoplasmic Reticulum
Source: Neuron. 2021 Jan 20;109(2):299–313.e9. doi: 10.1016/j.neuron.2020.10.005 (PMC7837115; doi:10.1016/j.neuron.2020.10.005)
Supplement: Table S2. Oligonucleotides Used in This Study, Related to STAR Methods [file mmc3.docx]

**SUPPLEMENTAL TABLE S2 - Oligonucleotides used in this study**

| REAGENT or RESOURCE | SOURCE | IDENTIFIER |
| --- | --- | --- |
| Oligonucleotides | | |
| shRNA: PanRyR: ACATGGAGACCAAGTGCTT | (Wu et al., 2013) | N/A |
| shRNA: Fip200#1: GAGAGAACTTGTTGAGAAA | (Ganley et al., 2009) | N/A |
| shRNA: Fip200#2: ACATGAAGGCTCAGAGAAA | (Ganley et al., 2009) | N/A |
| shRNA: Rtn3#1: TATGTTGGGATTGCCCGGGAT | (Bastide et al., 2017) | N/A |
| shRNA: Rtn3#2: CGATGGTGCATGTCAACAA | Adapted from (Siddiqi et al., 2018) | N/A |
| shRNA: Rtn3#3: ACAAGGCCCTCAAACTCATTA | Sigma-Aldrich | TRCN0000314216 |
| shRNA: CCPG1#1: GCTGCAGAAAACCAGTATT | Biosettia shRNA design | N/A |
| shRNA: CCPG1#2: GGTCCTTAAGCAGTACTTA | Biosettia shRNA design | N/A |
| shRNA: CCPG1#3: GGAGTTTGTGAGACATCAT | Biosettia shRNA design | N/A |
| shRNA: FAM134A1#1: GAGTTGATCCAGAGGATGTA | Biosettia shRNA design | N/A |
| shRNA: FAM134A1#1: GCAGTGGGACGTTTAGCTACG | Block-iT RNAi designer | N/A |
| shRNA: FAM134B1#1: GAAGTCTCTTGGACTGACAAT | Adapted from (Khaminets et al., 2015) | N/A |
| shRNA: FAM134B1#2: CACAAGGATGACAGTGAATTA | Adapted from (Khaminets et al., 2015) | N/A |
| shRNA: ATL3#1: GCCCCAACTTTGATGGGAAATTGAA | Adapted from (Khaminets et al., 2015) | N/A |
| shRNA: ATL3#2: GGGGTACATCAGATATTCCGGGCAG | Adapted from (Khaminets et al., 2015) | N/A |
| shRNA: ATL3#3: GATTCGAGATTGGAGTTTCCCTTAT | Adapted from (Rismanchi et al., 2008) | N/A |
| shRNA: ATL3#4: GCCCACTGAAATGATGCTTTA | Adapted from (Rismanchi et al., 2008) | N/A |
| shRNA: TEX263#1: GCAAGGCAAGGAGATTTCTAC | Block-iT RNAi designer | N/A |
| shRNA: TEX263#2: GCTGATACAAGTGATGCAA | Biosettia shRNA design | N/A |
| shRNA: TEX263#3: GGGTGAGGAGTAATGATAA | Biosettia shRNA design | N/A |
| shRNA: TEX263#4: GACAAGATCCATTTCATGTGC | Block-iT RNAi designer | N/A |
| shRNA: Non-targeting control: ACATGGAGACCAAGTGCTT | (Royle et al., 2005) | N/A |
| Genotype primers:  CRE: TM63 (CCGGGCTGCCACGACCAA), TM64 (GGCGCGGCAACACCATTTTT) | N/A | N/A |
| Genotype primers:  ATG5: P47 GAATATGAAGGCACACCCCTGAAATG, P48 ACAACGTCGAGCACAGCTGCGCAAGG, P49 GTACTGCATAATGGTTTAACTCTTGC | N/A | N/A |
